# Analysis on the stochastic evolution process of low-carbon transformation for supplier groups in construction supply chain

**DOI:** 10.1371/journal.pone.0264579

**Published:** 2022-03-08

**Authors:** Yaohong Yang, Jing Dai, Yi Zeng, Ying Liu

**Affiliations:** 1 School of Water Conservancy, North China University of Water Resources and Electric Power, Zhengzhou, China; 2 The Henan Key Laboratory of Water Resources Conservation and Intensive Utilization in the Yellow River Basin, Zhengzhou, Henan, China; China University of Mining and Technology, CHINA

## Abstract

To achieve the goal of "emission peak and carbon neutrality", it is particularly important to accelerate the transformation of low-carbon production as the construction industry of China’s major carbon emission. Considering the national industrial management system, this paper constructs a stochastic game model of supplier group’s production strategy in construction supply chain based on Moran process, analyzes the conditions for low-carbon emission strategy to fixate in supplier populations and become an evolutionary stable strategy, then, carries out numerical analysis on fixation probability and fixation times, and the influence of various factors on the evolution process is discussed, such as the initial willingness of suppliers to choose low-carbon emission strategy, the cost subsidy coefficient of government to low carbon suppliers, the reward and punishment of government and the credibility of suppliers. The results show that on the basis of vigorously promoting environmental protection, the government should implement a differentiated treatment policy through the establishment of carbon emissions standards, cost subsidies, incentives and punishment measures, and information disclosure systems to guide supplier groups to transformation into low-carbon production.

## 1 Introduction

In 2020, at the 75th General Debate of the United Nations General Assembly, China proposed to adopt stronger policies and measures to peak CO_2_ emissions by 2030 and strive to achieve carbon neutrality by 2060 [1]. China’s goal of "emission peak and carbon neutrality" has attracted strong attention from various industries once it was proposed. According to statistics, carbon dioxide emissions from construction industry account for more than 30% of total human greenhouse gas emissions [2]. In China, the construction industry ranks second in terms of carbon emissions, and it is crucial to promote the low-carbon transformation (LCT) of the construction industry [3–5]. Therefore, how to guide construction industry enterprises to low-carbon production as soon as possible and realize the low-carbon operation of the whole chain of the construction supply chain (CSC) has become an urgent issue to be solved.

The main sources of carbon emissions in CSC include: construction materials and components production, transportation process; equipment production and transportation process; engineering construction site production process, and engineering operation process. The suppliers involved in process include construction material manufacturers, component manufacturers, equipment manufacturers, transportation enterprises, construction enterprises, subcontracting enterprises, operation and maintenance enterprises, etc. The carbon emissions production strategy choices of enterprises determine the carbon emissions of CSC. The research on carbon emissions of CSC mainly focuses on carbon emissions calculation [6–8] and carbon emissions mechanism analysis of engineering construction [9–12] etc. To realize the low-carbon operation of CSC, the joint efforts of government management [13–15] and industry enterprises [16] are needed. On the one hand, government management needs to create institutional environment and take regulatory measures to guide enterprises to low-carbon production; on the other hand, enterprises need to take technical and management measures to reduce carbon emissions, such as using clean energy, reducing energy consumption through technological upgrading, installing carbon capture technology equipment, adopting green transportation methods, and adopting low-carbon construction technologies and techniques.

After double carbon target was proposed, the pressure for low-carbon transformation in the construction industry has increased abruptly, and the development of green and low carbon buildings has become mainstream direction [17]. According to the whole life cycle process of building products, the green supply chain management stages of construction industry can be divided into six stages: green planning, green design, green procurement, green construction, green operation and maintenance, and green end-of-life [18, 19]. The energy consumed and carbon emissions generated during the use of different building materials vary, and there is uncertainty impact on environment [20]. Some scholars have studied the carbon emissions in various stages of construction, such as, a multi-objective green design model for supertall buildings was proposed for minimizing CO_2_ emissions and costs in the construction and design phases of supertall buildings mega columns [21]; quantitative evaluation of sustainable development performance of building construction enterprises [22], etc. The achievement of low carbon goals is determined by joint efforts of enterprises, consumers, government and other subjects [23]. Factors such as the cost and efficiency of emission reduction of enterprises [24], the level of social responsibility of enterprises [25], the price and cost of low-carbon products [26], and government subsidies for low-carbon products influence whether enterprises decide to make the strategic choice of producing low-carbon products [27]. Consumer preferences for low-carbon products and the cost of carbon emission reduction investments influence the production decisions [28, 29] and product pricing decisions [30] of firms in the autonomous low-carbon supply chain. To achieve China’s carbon reduction targets, it has become a trend for government to take measures to limit the carbon emissions of firms [31]. Carbon subsidies and the level of corporation social responsibility implementation can contribute to emission reductions and improve overall supply chain performance [32, 33]. The government should increase consumers’ low-carbon preferences by setting more reasonable consumer subsidy rates [34, 35] etc. Some scholars have found that an increase in carbon taxes can motivate firms to reduce their carbon emissions [36]. From the perspective of implementation effectiveness, cost, and business acceptance, the environmental costs for manufacturers under a progressive carbon tax policy are smaller and should be prioritized [37]. The government can encourage companies to participate in R&D cooperation on emission reduction by providing tax breaks and special funds for R&D on emission reduction [38].

Some scholars also studied the carbon emission game between government and enterprises from the perspective of engineering construction management. Lu et al. have found that government environmental regulation and the extra technical cost of low-carbon production are the factors affecting the low-carbon transformation of construction enterprises. The greater the intensity of government environmental regulation, the lower the additional technical cost of low carbon production, the more inclined construction enterprises to low carbon production [39]. The specific situation for government to formulate and implement carbon emission reduction policies is that the central environmental protection department is responsible for formulating carbon emission reduction quotas and inspection standards, the local environmental protection department is responsible for quota allocation and environmental inspection, and the central environmental protection department supervises and evaluates the policy implementation of the local environmental protection department in China [40]. Local environmental protection departments are directly led by local governments in terms of administrative affiliation and funding allocation. Local governments’ pursuit of economic performance will require environmental protection departments to relax environmental supervision. Local environmental protection departments will have to cooperate with local governments’ decisions for political promotion and departmental interests, and carbon emission reduction performance will eventually be weakened [41]. The study also found that third-party oversight influences local government regulation and corporate carbon emissions [42, 43].

All these literatures consider suppliers as an individual in game model. In fact, the suppliers of CSC form finite populations, and suppliers are independent individuals with the freedom of decision making of production strategies, while the production strategies of suppliers are mutually influenced, so it is necessary to describe the production strategy evolution process of the supplier groups. In this paper, we use Moran process to study the stochastic evolution process of production strategy of suppliers groups.

On the one hand, research on Moran process is theoretical. Traulsen et al. summarized the Moran process in three steps: selection, reproduction and replacement [44], and Taylor et al. compared stochastic evolutionary game model for finite populations with replicative dynamic model for infinite populations to analyzed the connections and differences between the two [45]. Different from deterministic evolutionary games, stochastic evolutionary games introduces selection intensity. According to the dependence between fitness and individual payoffs, the selection is divided into strong selection and weak selection [46]. The dependence has two mapping forms: linear mapping and exponential mapping [47]. The update mechanism of the strategy on Moran process is an asynchronous update mechanism. When the player’s strategy has no mutation, the evolutionary process is a Markov chain with absorbing states, and the indicators for judging overall evolutionary dynamics are fixation probability and fixation time; when the player’s strategy has mutation, the strategic updating process is a Markov chain without absorbing states, which is judged by average abundance when strategy reaches a smooth distribution [48]. On the other hand, there are applied studies of Moran process, such as for the manufacturing industry, to analyze favorable conditions for the predominance of profit-maximizing production strategies and revenue-maximizing production strategies for a finite number of manufacturers [49], credit information sharing strategies for e-commerce platforms [50], the evolutionary process of strategies for whether consumers participate in crowd-funding [51], and the evolutionary process of quality improvement input decisions by production companies [52]; for social management, to analyze the process of strategy evolution for trainee attacks under de-radicalization approach [53], the process of strategy evolution for attacks by separatist terrorist organizations [54], the mechanism of defusing mass emergencies [55], and strategic evolution process for counter-terrorism by the governments of member countries of anti-terrorism coalition [56]; and for engineering management, to analyze the favorable conditions for promoting the strategy of not adopting opportunistic behavior by contractors of PPP projects [57].

Low-carbon production has become an inevitable choice for the development of construction industry at present. And achieving the goal requires not only the efforts of the suppliers themselves, but also the strong support of government. From the supplier’s point of view, each supplier faces two choices: low-carbon emission strategy and high-carbon emission strategy. Choosing low-carbon emission strategy means making technological improvements, process upgrades, etc., which requires more product costs. It obviously requires government’s guidance and intervention. From the government’s point of view, in order to guide suppliers to switch from existing production mode to low-carbon production mode, and to promote switch as soon as possible, it is necessary to develop a subsidy system, reward and punishment system and reputation system, etc. Thus, it is necessary to study the selection process of low-carbon transition strategies for supplier groups.

The remainder of this paper is organized as follows. In Section 2, based on Moran process, we construct a stochastic evolutionary model of carbon emissions strategies of suppliers in CSC and analyze evolutionary trend of supplier’s choice of low-carbon strategies. In Section 3, the main factors affecting supplier’s low-carbon strategy choice by calculating fixation probability under strong selection and weak selection is analyzed. In Section 4, the fixation time under neutral selection and weak selection is calculated. In Section 5, we analyze the influence of changes in parameters such as supplier’s initial willingness, government’s subsidies, government’s rewards and punishments, and supplier’s reputation on evolutionary process based on fixation probability under strong and weak selection; conditional fixation time and unconditional average fixation time under weak selection. The conclusions and its implications are shown in Section 6.

## 2 Stochastic game model

Supplier’s LCT affects the process of achieving dual-carbon goals of construction industry. In fact, suppliers are finite populations [58]. The behavior of each supplier affects the evolution of supplier group’s behavior. It is meaningful to find the threshold value scale of low-carbon strategy as an evolutionary stable strategy through model, and analyze the influence process of related variables on the evolution process of supplier group’s behavior. We establish a stochastic evolutionary game model and consider 2*2 strategies in our study. The basic assumptions and related parameters of study are setting as follows.

### 2.1 Basic assumptions and parameter settings

#### Assumption 1

There is stochasticity in strategy choice of suppliers. Enterprises with high carbon emissions may be forced to choose labeling low carbon emission strategy under environmental pressure. Enterprises with low carbon emissions may choose excessive carbon emission behavior due to high cost pressure and force majeure. Uncertainties such as technological advancement, policy orientation, risk preference, and blind herd mentality can affect supplier’s strategy choices.

#### Assumption 2

There are N suppliers in market. Suppliers constantly adjust their own strategies through experience accumulation, information exchange, imitation learning and so on. When one strategy dominates, another strategy will be replaced. It can be seen as a random birth and death process.

#### Assumption 3

Supplier enterprises are in a complex market. Whether they choose active or passive carbon emission reduction, due to regional differences, government’s policies, changes in consumer demand and other factors, their strategic selection process is dynamic, complex and random. Generally, enterprises choose strategies according to their direct and potential benefits. Due to the large differences in carbon emission reduction costs in different regions, enterprises in the region have different emission reduction potentials and potential benefits of carbon emissions. Each enterprise in the region chooses different carbon emission strategies. Focusing on the index of carbon emissions, no matter which strategy the enterprise initially chooses, the final result is that carbon emissions are too high or too low. This paper calls it high carbon emission strategy and low carbon emission strategy [59]. Abstract the supplier groups adopting low-carbon emission strategy and the supplier groups adopting high-carbon emission strategy into two players adopting different strategies: Strategy Y, which is low-carbon emission strategy and strategy S, which is high-carbon emission strategy. Low-carbon emission strategy implies that suppliers adopt positive attitude to reduce carbon emissions, such as: material and equipment manufacturers make technological improvements, use low energy technology or increase carbon capture equipment, and construction companies choose green construction techniques. High-carbon emission strategy implies that suppliers adopt wait-and-see attitude and use original process and technology to produce.

#### Assumption 4

Government subsidies have a direct or indirect impact on corporate decision-making, corporate profits, green building products and the overall environmental benefits of the construction supply chain. On the one hand, government subsidies reduce corporate financing constraints, on the other hand, reduce the industry access threshold. According to the supplier’s R&D investment in emission reduction, government gives certain subsidies to suppliers adopting low-carbon strategies. And government may subsidize the R&D cost of construction products, or subsidize the fixed production cost of products. Either form of subsidy, the supplier’s production costs is appropriately reduced. The amount of government subsidies is determined by government’s cost subsidy coefficient and the production cost of suppliers.

#### Assumption 5

Government sets a standard value for carbon emissions of various suppliers. When the actual carbon emissions from suppliers are greater than standard value, government punishes suppliers by raising carbon taxes; when the actual carbon emissions of suppliers are less than the standard value, government will reward the suppliers. The rewards and punishments are determined by the product of the difference between actual carbon emissions and standard carbon emissions. The reward and punishment is determined by a combination of reward and punishment and the product of the difference between actual carbon emissions and standard carbon emissions.

#### Assumption 6

The conventional supervision method commonly used in construction industry is notification and inspection. It often has regularity, the participating units through the law to find inspection blind spots resulting in a significant reduction in government oversight. Unannounced inspection is a new inspection method in construction industry. An unannounced inspection system means that inspector’s sudden inspection on the inspected. Compared with the traditional regular fixed-point quality inspection mode, it can effectively avoid moral hazard and improve the incentive effect. And from the way of quality sampling, its randomness and confidentiality are further strengthened. It helps the inspector find more situations as the inspected really are and leads to a reduction of the regulatory burden. Similar to unannounced inspection system implemented by construction industry [60], government adopts ’’Double random and one public’’ method to check the carbon emissions reduction behavior of suppliers. ’’Double random’’ refers to random selection of inspectors, random selection of the inspected. ’’One public’’ refers to government discloses the results of carbon emissions inspection. The supplier’s social reputation will be affected when government discloses inspection result.

#### Assumption 7

When suppliers choose low carbon strategy, green building product supply increases; the demand side subjects also increase the demand for green building products due to low-carbon consumption preference, green sensitivity, market environment and other factors. The pressure on government to regulate the market environment of green construction is also reduced, and the social recognition of the construction industry is increased. When suppliers choose high carbon strategy, the industry recognition is reduced.

The relevant parameter settings are shown in Table 1.

**Table 1:** Related parameter settings and its meanings.

Related parameters	Meanings
*Q*	Basic benefits for suppliers
*C*_1_	The cost of suppliers choosing low-carbon emission strategy, including new technology, new material, training cost, etc.
*C*_2_	The cost of suppliers choosing high-carbon emission strategy
*a*	Initial willingness of suppliers to choose low-carbon emission strategy
*α*	The government’s cost subsidy coefficient for suppliers who choose low-carbon emission strategy
*k*	The government’s incentives and penalties coefficient for suppliers
*L*	Government mandated supplier’s standard carbon emissions
*B*_1_	Actual carbon emissions when suppliers choose low-carbon emission strategy
*B*_2_	Actual carbon emissions when suppliers choose high-carbon emission strategy
*r*	Reputational impact on suppliers as a result of public government unannounced carbon emission inspections
*G*	Public acceptance of the construction industry as a whole rises when both sides choose low-carbon emission strategy
*D*	Public acceptance of the construction industry as a whole declines when both sides choose high-carbon emission strategy

Where the cost paid by suppliers choosing low-carbon emission strategy is greater than the cost paid by suppliers choosing high-carbon emission strategy, i.e., *C*_1_ > *C*_2_. The initial willingness of suppliers to choose low-carbon emission strategy is *a*, a∈[0,1].*a* → 1, suppliers tend to choose strategy Y, considering suppliers as risk-preferred; *a* → 0, suppliers tend to choose strategy S, considering suppliers as risk-averse. The carbon emissions of suppliers choose low-carbon emission strategies are smaller than the standard carbon emissions stipulated by government (*B*_1_ < L), and the carbon emissions of suppliers choosing high-carbon emission strategies are larger than the standard carbon emissions of products stipulated by government (*L* < *B*_2_).

The payoffs matrix of supplier choose different strategies is shown in Table 2.

**Table 2:** Game payoffs matrix under the supplier choice carbon emissions strategy.

Strategy Selection		Supplier 2	
		Strategy Y	Strategy S
Supplier 1	Strategy Y	Q − *a*C_1_ + *α*C_1_ + *k* (*L* − *B*_1_) + *r* + G Q – *a*C_1_ + *α*C_1_ + *k* (*L* − *B*_1_) + *r* + G	Q − *a*C_1_ + *α*C_1_ + *k* (*L* − *B*_1_) + *r* Q − (1 − *a*)C_2_ − *k* (*B*_2_ − *L*) − *r*
	Strategy S	Q − (1 − *a*)C_2_ − *k*(*B*_2_ − *L*) − *r*	Q − (1 − *a*)C_2_ − *k*(*B*_2_ − *L*) − *r* − *D*
		Q − *a*C_1_ + *α*C_1_ + *k* (*L* − *B*_1_) + *r*	Q − (1 − *a*)C_2_ − *k*(*B*_2_ − *L*) − *r* − *D*

### 2.2 Model solving

According to Table 2, the expected payoffs for supplier selection strategy Y and strategy S are calculated as follows, respectively.

$$E_{i}^{Y}=\frac{i-1}{N-1}[Q-aC_{1}+\alpha C_{1}+k(L-B_{1})+r+G]+\frac{N-i}{N-1}[Q-aC_{1}+\alpha C_{1}+k(L-B_{1})+r]$$ (1)

$$E_{i}^{S}=\frac{i}{N-1}[Q-(1-a)C_{2}+k(B_{2}-L)-r]+\frac{N-i-1}{N-1}[Q-(1-a)C_{2}+k(B_{2}-L)-r-D]$$ (2)

where *i* = 1,2,…,N–1, *i* is the number of suppliers choosing strategy Y and *N*-*i* is the number of suppliers choosing strategy S.

Compared to linear mapping, exponential mapping change the speed of process, allowing for greater changes in selection intensity [47], and qualitative results do not change depending on the form of the mapping, so the fitness is assumed to be an exponential function of the gain, where ω ≥ 0.

$$f_{i}^{Y}=e^{\omega E_{i}^{Y}}$$ (3)

$$f_{i}^{S}=e^{\omega E_{i}^{S}}$$ (4)

The probability that the suppliers choosing strategy Y replicate and increase by one is $\frac{if_{i}^{Y}}{if_{i}^{Y}+(N-i)f_{i}^{S}}$. As time step evolves, the number of suppliers adopting strategy Y may increase by one, decrease by one, or remain constant [61]. The stochastic dynamic process can be represented by a third-order diagonal matrix, i.e., a probability transfers matrix. In the probability transfers matrix, all elements except the diagonal are zero in probability transfers matrix. The elements on diagonal are calculated as follows.

$$P_{i\to i+1}=\frac{if_{i}^{Y}}{if_{i}^{Y}+(N-i)f_{i}^{S}}*\frac{N-i}{N}$$ (5)

$$P_{i\to i-1}=\frac{(N-i)f_{i}^{S}}{if_{i}^{Y}+(N-i)f_{i}^{S}}*\frac{i}{N}$$ (6)

$$P_{i\to i}=1-P_{i\to i+1}-P_{i\to i-1}$$ (7)

There are two stable states in evolutionary process: *i* = 0, all suppliers adopt strategy S, i.e., choose high-carbon emission strategy; *i* = *N*, all suppliers adopt strategy Y, i.e., choose low-carbon emission strategy. If supplier populations present one of these states, supplier populations keep the state stable.

Denote *x*_*i*_ as the probability that the supplier of the selection strategy Y evolves from the initial state of *i* individuals selected to all N individuals taken. According to full probability formula, the probabilities of convergence to two stable states of *x*_0_ = 0 and *x*_*N*_ = 1 are

$$x_{i}=x_{i+1}P_{i\to i+1}+x_{i}P_{i\to i}+x_{i-1}P_{i\to i-1}$$ (8)

Substituting Eqs (5) ~ (7) into Eq (8), we get

$$x_{i}=\frac{1+\sum_{j=1}^{i-1}\prod_{k=1}^{j}\frac{f_{k}^{S}}{f_{k}^{Y}}}{1+\sum_{j=1}^{N-1}\prod_{k=1}^{j}\frac{f_{k}^{S}}{f_{k}^{Y}}}$$ (9)

Only one supplier chooses strategy Y, the probability that the final strategy Y is stable is fixation probability denoted as *ρ*_*Y*_. Substituting *i* = 1 into Eq (9), we get

$$\rho_{Y}=x_{1}=\frac{1}{1+\sum_{j=1}^{N-1}\prod_{k=1}^{j}e^{\omega [E_{k}^{S}-E_{k}^{Y}]}}=\frac{1}{1+\sum_{j=1}^{N-1}exp\left[\omega \sum_{k=1}^{j}\left(E_{k}^{S}-E_{k}^{Y}\right)\right]}$$ (10)

Only one supplier chooses strategy S, the probability that the final strategy S is stable is fixation probability denoted as *ρ*_*S*_.

$$\rho_{S}=1-x_{N-1}=\frac{1}{1+\sum_{j=1}^{N-1}\prod_{k=j}^{N-1}e^{\omega [E_{k}^{Y}-E_{k}^{S}]}}=\frac{1}{1+\sum_{j=1}^{N-1}exp\left[\omega \sum_{k=1}^{j}\left(E_{k}^{Y}-E_{k}^{S}\right)\right]}$$ (11)

The ratio of probability of strategy Y to strategy S fixate is

$$\frac{\rho_{Y}}{\rho_{S}}=\frac{1+\sum_{j=1}^{N-1}exp\left[\omega \sum_{k=1}^{j}\left(E_{k}^{Y}-E_{k}^{S}\right)\right]}{1+\sum_{j=1}^{N-1}exp\left[\omega \sum_{k=1}^{j}\left(E_{k}^{S}-E_{k}^{Y}\right)\right]}=\prod_{j=1}^{N-1}\frac{f_{j}^{Y}}{f_{j}^{S}}=exp\left[\omega \sum_{j=1}^{N-1}\left(E_{j}^{Y}-E_{j}^{S}\right)\right]$$ (12)

Without considering strategic mutation, the player with higher fixation probability is more likely to become an evolutionary stable strategy. That is, when *ρ*_*Y*_ > *ρ*_*S*_, the suppliers has a high probability of choosing strategy Y for a considerable period of time, and strategy Y is more likely to become an evolutionary stable strategy; when *ρ*_*Y*_ < *ρ*_*S*_, strategy S has a higher probability of becoming an evolutionary stable strategy [45].

## 3 Fixation probability

### 3.1 Strong selection

When suppliers make rational decisions, evolutionary process depends entirely on expected payoffs, i.e., it is a strong selection process when *ω* → ∞. By comparing the relationship between $f_{i}^{Y}$ and $f_{i}^{S}$ in each state *i*, we determine whether the number of suppliers choose strategy Y increases or decreases in state *i*.

Let

$$h_{i}=f_{i}^{Y}-f_{i}^{S},i=1,2,3,\ldots .N-1$$ (13)

#### Theorem 1

If *h*_1_ > 0, The supplier’s choose behavior support strategy Y to invade strategy S. If *h*_*N*– 1_ < 0, The supplier’s choose behavior support strategy S to invade strategy Y [45].

Substituting Eqs (1) ~ (4) into Eq (13), we get

$$h_{1}=f_{1}^{Y}-f_{1}^{S}=e^{\omega [Q-aC_{1}+\alpha C_{1}+k(L-B_{1})+r]}-e^{\omega [Q-(1-a)C_{2}-k(B_{2}-L)-r-\frac{N-2}{N-1}D]}$$ (14)

$$h_{N-1}=f_{N-1}^{Y}-f_{N-1}^{S}=e^{\omega [Q-aC_{1}+\alpha C_{1}+k(L-B_{1})+r+\frac{N-2}{N-1}G]}-e^{\omega [Q-(1-a)C_{2}-k(B_{2}-L)-r]}$$ (15)

By Theorem 1, the following scenarios may exist.

- *h*_1_ > 0, the number of suppliers taking strategy Y is growing, which favors invasion strategy S. *h*_*N*– 1_ < 0, the number of N-1 suppliers taking strategy Y is decreasing, which favors strategy S to invade strategy Y.
- If both *h*_1_ > 0 and *h*_*N*– 1_ > 0 are satisfied, it is in favor of strategy Y to replace strategy S. It is against strategy S to replace strategy Y, and strategy Y becomes an evolutionary stable strategy.
- If both *h*_1_ > 0 and *h*_*N*– 1_ < 0 or *h*_1_ < 0 and *h*_*N*– 1_ > 0 are satisfied, the selection is in favor of strategy Y and strategy S replacing each other, and the hybrid strategy is an evolutionary stable strategy.

Rectifying Eqs (14) and (15), the following proposition is obtained.

#### Proposition 1

When -*aC*_1_ + *αC*_1_ + *k*(*L*–*B*_1_) + *r* > -(1–*a*)*C*_2_ –*k*(*B*_2_–*L*)–*r*, forN ≥ 2, there is *h*_1_ > 0 and *h*_*N*– 1_ > 0. The supplier’s strategy selection behavior supports strategy Y replacing strategy S and strategy Y prevails.

Proof: *h*_1_ > 0 is equivalent to $E_{1}^{Y}>E_{1}^{S}$, and *h*_*N*– 1_ > 0 is equivalent to $E_{N-1}^{Y}>E_{N-1}^{S}$. By the basic assumptions *C*_1_ > *C*_2_ and *B*_1_ < *L* < *B*_2_, combined with the properties of the exponential function, the finishing of Eqs (14) and (15) shows that when -*aC*_1_ + *αC*_1_ + *k*(*L*–*B*_1_) + *r* > -(1–*a*)*C*_2_ –*k*(*B*_2_–*L*)–*r*, *h*_1_ > 0 and *h*_*N*– 1_ < 0. Therefore, the conclusion of Proposition 1 holds.

Proposition 1 shows that supplier’s strategic choice is related to factors such as the supplier’s initial willingness, government’s cost subsidies, government’s rewards and punishments, and supplier’s reputation. When supplier chooses low-carbon emission strategy, the benefits they get are greater than that of high-carbon emission strategy, supplier’s selection behavior support strategy Y invades strategy S, and low-carbon emission strategy becomes supplier’s evolutionary stable strategy.

#### Proposition 2

When supplier makes a strategy choice based on expected payoffs, -*aC*_1_ + *αC*_1_ + *k*(*L*–*B*_1_) + *r* > -(1–*a*)*C*_2_ –*k*(*B*_2_–*L*)–*r* when $1+\sum_{j=1}^{N-1}exp\left[\omega \sum_{k=1}^{j}\left(E_{k}^{S}-E_{k}^{Y}\right)\right]<N<1+\sum_{j=1}^{N-1}exp[\omega \sum_{k=1}^{j}\left(E_{k}^{Y}-E_{k}^{S}\right)]$, there exists a threshold value, $N_{0}=\max(\frac{-aC_{1}+\alpha C_{1}+k(L-B_{1})+2G+2r+(1-a)C_{2}+k(B_{2}-L)}{-aC_{1}+\alpha C_{1}+k(L-B_{1})+G+2r+(1-a)C_{2}+k(B_{2}-L)}$, $\frac{-aC_{1}+\alpha C_{1}+k(L-B_{1})-(1-a)C_{2}+k(B_{2}-L)+2r+2D}{-aC_{1}+\alpha C_{1}+k(L-B_{1})+2r+(1-a)C_{2}+k(B_{2}-L)+D}$. When *N* ≥ *N*_0_, strategy Y becomes an evolutionary stable strategy. There also exists a threshold value $N_{1}=\min(\frac{-aC_{1}+\alpha C_{1}+k(L-B_{1})+2G+2r+(1-a)C_{2}+k(B_{2}-L)}{-aC_{1}+\alpha C_{1}+k(L-B_{1})+G+2r+(1-a)C_{2}+k(B_{2}-L)}$$\frac{-aC_{1}+\alpha C_{1}+k(L-B_{1})-(1-a)C_{2}+k(B_{2}-L)+2r+2D}{-aC_{1}+\alpha C_{1}+k(L-B_{1})+2r+(1-a)C_{2}+k(B_{2}-L)+D}$. When *N* ≤ *N*_1_, strategy S becomes an evolutionary stable strategy. If $\text{N}\in {[N}_{1}{,N}_{0}\text{]}$, the mixed equilibrium of two strategies becomes evolutionary stable strategy with a mixing ratio of ($\frac{1}{N}$, $\frac{N-1}{N}$). The suppliers all choose strategy Y if the number of individuals choosing strategy Y is greater than some threshold value.

Proof: If $\rho_{Y}>\frac{1}{N}$, i.e., $1+\sum_{j=1}^{N-1}exp\left[\omega \sum_{k=1}^{j}\left(E_{k}^{S}-E_{k}^{Y}\right)\right]<N$, supplier’s choice of support strategy Y invades strategy S. $\rho_{S}<\frac{1}{N}$, i.e., $1+\sum_{j=1}^{N-1}exp\left[\omega \sum_{k=1}^{j}\left(E_{k}^{Y}-E_{k}^{S}\right)\right]>N$, supplier’s choice of resistance strategy S invasion strategy Y, finishing with, $1+\sum_{j=1}^{N-1}exp\left[\omega \sum_{k=1}^{j}\left(E_{k}^{S}-E_{k}^{Y}\right)\right]<N<1+\sum_{j=1}^{N-1}exp\left[\omega \sum_{k=1}^{j}\left(E_{k}^{Y}-E_{k}^{S}\right)\right]$. Prove that *h*_1_ > 0 i.e., prove that $Q=aC_{1}+\alpha C_{1}+k(L-B_{1})+r>Q-(1-a)C_{2}-k(B_{2}-L)-r-\frac{N-2}{N-1}D$. Making it equal to 0, the collation yields, $\text{N}=\frac{-aC_{1}+\alpha C_{1}+k(L-B_{1})-(1-a)C_{2}+k(B_{2}-L)+2r+2D}{-aC_{1}+\alpha C_{1}+k(L-B_{1})+2r+(1-a)C_{2}+k(B_{2}-L)+D}$. Prove that *h*_*N-*1_ > 0 i.e., prove that $Q=aC_{1}+\alpha C_{1}+k(L-B_{1})+r+\frac{N-2}{N-1}G>Q-(1-a)C_{2}-k(B_{2}-L)-r$. Make it equal to 0 and collate to get, $N=\frac{-aC_{1}+\alpha C_{1}+k(L-B_{1})+2G+2r+(1-a)C_{2}+k(B_{2}-L)}{-aC_{1}+\alpha C_{1}+k(L-B_{1})+G+2r+(1-a)C_{2}+k(B_{2}-L)}$, Proposition 2 is proved.

Proposition 2 shows that when suppliers make strategic choices based entirely on expected payoffs, there is a threshold value for supplier population’s size. When the population’s size is small, the supplier chooses strategy S, that is, high-carbon emission strategy. When the population’s size is greater than a certain threshold value, the supplier will choose strategy Y, that is, choose low-carbon emission strategy. It shows that supplier has a large scale effect in strategic selection process.

### 3.2 Weak selection

The supplier’s strategic choice not only depends on expected payoffs, but also affected by stochastic factors such as policy orientation, low-carbon technology improvement, subject preference, and blind herding psychology. Taking neutral invasion probability 1/*N* as the benchmark, the evolution process of supplier under weak selection is studied, and supplier’s final strategic choice is obtained. That is, *ω* → 0.

#### Theorem 2

If *ρ*_*Y*_ > 1/*N*, supplier’s choose behavior support strategy Y to invade strategy S; if *ρ*_*Y*_ < 1/*N*, supplier’s choose behavior oppose strategy Y to invade strategy S [45].

Expanding Eqs (10) ~ (11) using Taylor’s formula yields

$$\rho_{Y}=\frac{1}{1+\sum_{j=1}^{N-1}\prod_{k=1}^{j}\frac{f_{S}}{f_{Y}}}\approx \frac{1}{N}+\frac{\omega }{6N}(a_{1}+a_{2}N)$$ (16)

$$\rho_{S}=\frac{1}{1+\sum_{j=1}^{N-1}\prod_{k=j}^{N-1}\frac{f_{Y}}{f_{S}}}\approx \frac{1}{N}+\frac{\omega }{6N}(a_{3}+a_{4}N)$$ (17)

where *a*_1_ = 3*aC*_1_−3*αC*_1_ – 3*k*(*L*–*B*_1_)– 6*r* – 2*G* – 3(1-*a*)*C*_2_ – 3*k*(*B*_2_–*L*)– 4*D*, *a*_2_ = -[3*aC*_1_−3*αC*_1_ - 3*k*(*L*–*B*)– 6*r* –*G*– 3(1- *a*)*C*_2_ – 3*k*(*B*_2_–*L*)- 2*D*], *a*_3_ = -[3*aC*_1_−3*αC*_1_ - 3*k*(*L*–*B*_1_)– 6*r* – 4*G* – 3(1- *a*)*C*_2_ – 3*k*(*B*_2_–*L*)- 2*D*], *a*_4_ = -3*aC*_1_−3*αC*_1_ - 3*k*(*L*–*B*_1_)– 6*r* – 2*G* – 3(1- *a*)*C*_2_ – 3*k*(*B*_2_–*L*)- 2*D*.

#### Proposition 3

Under the condition of weak selection, the expected payoffs have a very small impact on supplier’s strategic selection. For *N* ≥ 2, if it satisfies -3*aC*_1_ + 3*αC*_1_ + 3*k*(*L*–*B*_1_) + 6*r* + 2*G* > −3(1-*a*)*C*_2_ – 3*k*(*B*_2_–*L*)– 4*D*, then $\rho_{Y}>\frac{1}{N}$, $\rho_{S}>\frac{1}{N}$.

Proof: $\rho_{Y}>\frac{1}{N}$ is equivalent to *a*_1_ + *a*_2_ *N* > 0. When -[3*aC*_1_−3*αC*_1_ - 3*k*(*L*–*B*_1_)– 6*r* – 2*G* – 3(1- *a*)*C*_2_ – 3*k*(*B*_2_–*L*)- 4*D*]>0, $\frac{\partial (a_{1}+a_{2}N)}{\partial \text{N}}>0$. When N = 2, *a*_1_ + *a*_2_*N* > 0, the proposition is proved.

Proposition 3 shows that when the benefits of a supplier choosing low-carbon emission strategy is greater than its benefits of choosing high-carbon emission strategy, the choice favors strategy Y invading strategy S, Strategy Y prevails. When the benefits from supplier’s choice of low-carbon emission strategy are not large enough, the supplier will not actively choose low-carbon emission strategy, and because the cost of supplier’s choice of low-carbon emission strategy is too large, it will choose to produce using the original process because of speculation. It shows that it is necessary for government to take incentives for suppliers, and appropriate incentives can enhance supplier’s motivation to choose low-carbon emission strategies.

In summary, whether it’s in strong selection or weak selection, supplier’s strategic choice is closely related to factors such as population’s size, the initial willingness and reputation of the supplier, government’s cost subsidies, and the intensity of rewards and punishments. In the situation of strong selection, the benefits of supplier choosing low-carbon emission strategy are greater than the benefits of choosing high-carbon emission strategy. The supplier’s choose behavior support strategy Y invades strategy S, and low-carbon emission strategy become an evolutionary stable strategy for supplier groups. When supplier’s population size is greater than a certain threshold value, low-carbon emission strategy will become an evolutionary stable strategy. Otherwise, the supplier will choose high-carbon emission strategy. In the situation of weak selection, when suppliers tend to choose high-carbon emission strategy, the benefits are less than it choose low-carbon emission strategy, the low-carbon emission strategy prevails.

## 4 Fixation time

Fixation time is another important metric in determining stochastic evolution process, contains unconditional average fixation time and conditional average fixation time [48], when there is no mutation in supplier’s strategic selection process. Unconditional average fixation time *t*_*i*_ is the average time for a mutant to reach any absorbing state from state *i*. Conditional average fixation time $t_{i}^{Y}$ or $t_{i}^{S}$ is the average time it takes for a mutant to reach absorbing state Y or S from state *i* and eventually reach only state Y or S [62]. The mutant here refers to the name of offspring that is bred, and doesn’t mean that the bred offspring use different strategies. That is to say, the supplier used low-carbon production strategy or high-carbon production strategy before, and the supplier selected for reproduction also uses low-carbon production strategy or high-carbon production strategy.

The expression for unconditional average fixation time is shown below with the boundary condition *t*_0_ = *t*_*N*_ = 0.

$$t_{i}=1+t_{i-1}P_{i\to i-1}+(1-P_{i\to i-1}-P_{i\to i+1})t_{i}+t_{i}+t_{i+1}P_{i\to i+1}$$ (18)

where

$$t_{i}=\sum_{k=i}^{N-1}\sum_{l=1}^{k}\frac{1}{P_{l\to l+1}}\prod_{m=l+1}^{k}\frac{P_{m\to m-1}}{P_{m\to m+1}}-t_{1}\sum_{k=i}^{N-1}\prod_{m=1}^{k}\frac{P_{m\to m-1}}{P_{m\to m+1}}$$ (19)

$$t_{1}=\delta_{1}^{Y}\sum_{k=1}^{N-1}\sum_{l=1}^{k}\frac{1}{P_{l\to l+1}}\prod_{m=l+1}^{k}\frac{P_{m\to m-1}}{P_{m\to m+1}}$$ (20)

$$\delta_{i}^{Y}=\frac{1+\sum_{k=1}^{i=1}\prod_{m=1}^{k}\frac{P_{m\to m-1}}{P_{m\to m+1}}}{1+\sum_{k=1}^{N-1}\prod_{m=1}^{k}\frac{P_{m\to m-1}}{P_{m\to m+1}}}$$ (21)

Taylor et al. noted that conditional average fixation time is same for both mutants [63], so when the supplier selects the state of strategy Y in its entirety, conditional average fixation time is

$$\delta_{i}^{Y}t_{i}^{Y}=\delta_{i-1}^{Y}P_{i\to i-1}(t_{i-1}^{Y}+1)+\delta_{i}^{Y}(1-P_{i\to i-1}-P_{i\to i+1})(t_{i}^{Y}+1)+\delta_{i+1}^{Y}p_{i\to i+1}(t_{i+1}^{Y}+1)$$ (22)

$$t_{i}^{Y}=\frac{1}{\delta_{i}^{Y}}\sum_{k=i}^{N-1}\sum_{l=1}^{k}\frac{\delta_{l}^{Y}}{p_{i\to i+1}}\prod_{m=l+1}^{k}\frac{p_{m\to m-1}}{p_{m\to m+1}}-t_{1}^{Y}=\frac{\delta_{1}^{Y}}{\delta_{i}^{Y}}\sum_{k=i}^{N-1}\prod_{m=1}^{k}\frac{p_{m\to m-1}}{p_{m\to m+1}}$$ (23)

$$t_{1}^{Y}=\sum_{k=1}^{N-1}\sum_{l=1}^{k}\frac{\delta_{l}^{Y}}{p_{i\to i+1}}\prod_{m=l+1}^{k}\frac{p_{m\to m-1}}{p_{m\to m+1}}$$ (24)

The specific expressions for fixation times under neutral selection and weak selection are as follows [64, 65]. In neutral selection situation, i.e., ω = 0, is substituted into Eqs (3) ~ (6), which yields $\frac{P_{i\to i-1}}{P_{i\to i+1}}=1$. Substituting into Eqs (15) ~ (19), it yields

$$t_{1}=\frac{1}{N}\sum_{k=1}^{N-1}\sum_{l=1}^{k}\frac{2N}{l(N-1)}=NH_{N-1}$$ (25)

$$H_{N-1}=\sum_{l=1}^{N-1}\frac{1}{l}$$ (26)

$$t_{1}^{Y}=\sum_{k=1}^{N-1}\sum_{l=1}^{k}\frac{1}{N}\prod_{m=l+1}^{k}\frac{2N^{2}}{l(N-l)}=N(N-1)$$ (27)

The formula for calculating fixation time in weak selection is complex, and refer to existing literature [65], unconditional average fixation time and conditional average fixation time for supplier to select low-carbon emission strategy to reach a steady state are

$$t_{1}=NH_{N-1}+e\frac{N}{2}(N+1-2H_{N})\omega$$ (28)

$${\text{t}}_{1}^{Y}=N(N-1)-f\frac{N^{2}(N^{2}-3N+2)}{36}\omega$$ (29)

where

$$e=\frac{[-aC_{1}+\alpha C_{1}+k(L-B_{1})+2r+(1-a)C_{2}+k(B_{2}-L)+D]N+aC_{1}-\alpha C_{1}-k(L-B_{1})-2r-(1-a)C_{2}-k(B_{2}-L)-D-G}{N-1}f=\frac{G-D}{N-1}$$

Then we use numerical simulation to analyze changes in parameters such as initial willingness and reputation of suppliers, government’s cost subsidies to low-carbon suppliers, government’s rewards and punishments to suppliers, and evolution time when supplier group’s strategy reaches homogeneous state process in weak selection, it will be shown in 5.3 and 5.4.

## 5 Numerical analysis

In the field of construction engineering in China, there is no policy document to define the threshold value of carbon emissions and a mature management system for carbon emissions, and here we analyze the strategy evolution process of supplier groups under different parameter changes through numerical simulation.

In terms of parameter settings in the simulation model, we refer to existing research in relevant literature to set each parameter values [28, 66, 67]: the basic benefit to suppliers is 20. The cost when supplier choose low-carbon emission strategy is 15, the cost of choosing high-carbon emission strategy is 12, the initial willingness of suppliers is s0.7, the government’s subsidy coefficient is 0.1, the government’s rewards and penalties coefficient is 0.2, the government’s standard carbon emission is 10, the carbon emissions when suppliers choose low-carbon emission strategy is 8, the carbon emissions when suppliers choose high-carbon emission strategy is 12. The supplier’s reputation impact value is 0.7. When suppliers all choose low-carbon emission strategy, the public’s recognition of industry rise value is 0.7. And suppliers all choose high-carbon emission strategy, the public’s recognition of industry decline value is 2. ω → *∞* in strong selection situation, and let ω = 1 for calculation. ω = 0.001 in weak selection situation.

The initial willingness of suppliers, cost subsidy coefficient, government’s rewards and punishments, and supplier’s reputation are used as variables, and variables are valued according to different situations. The evolution trend of supplier group is discussed through two indicators of fixation probability and fixation time.

### 5.1 Strong selection

#### 5.1.1 The influence of supplier’s initial willingness

With other parameters unchanged, discuss the evolution process of supplier populations when supplier’s initial willingness changes.

**Fig 1:**
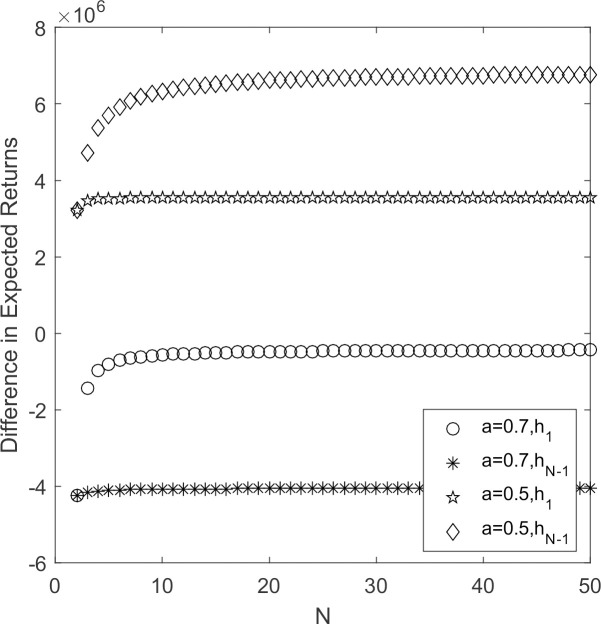
*h*_1_/*h*_N-1_~*a* change chart.

As can be seen from Fig 1, in the situation of constant government’s cost subsidy, reward and punishment, *a* = 0.7, *h*_1_ < 0 and *h*_*N*-1_ < 0. According to Theorem 1, supplier’s choice behavior resistance strategy Y invasion strategy S, support strategy S invasion strategy Y, and high-carbon emission strategy prevails. *a* = 0.5, supplier’s choice behavior support strategy Y invasion strategy S, resistance strategy S invasion strategy Y, and low-carbon emission strategy prevails. When t government has certain subsidies, rewards and punishments for suppliers, suppliers choose low-carbon emission strategy means pay more costs. In strong selection when supplier is completely rational, strategic choice is determined by expected payoffs, the cost becomes large, no corresponding revenue, supplier populations will choose high-yield high-carbon strategy.

#### 5.1.2 Effect of government’s subsidy factor

With other parameters unchanged, discuss the evolution process of supplier populations when government’s subsidy coefficient changes.

**Fig 2:**
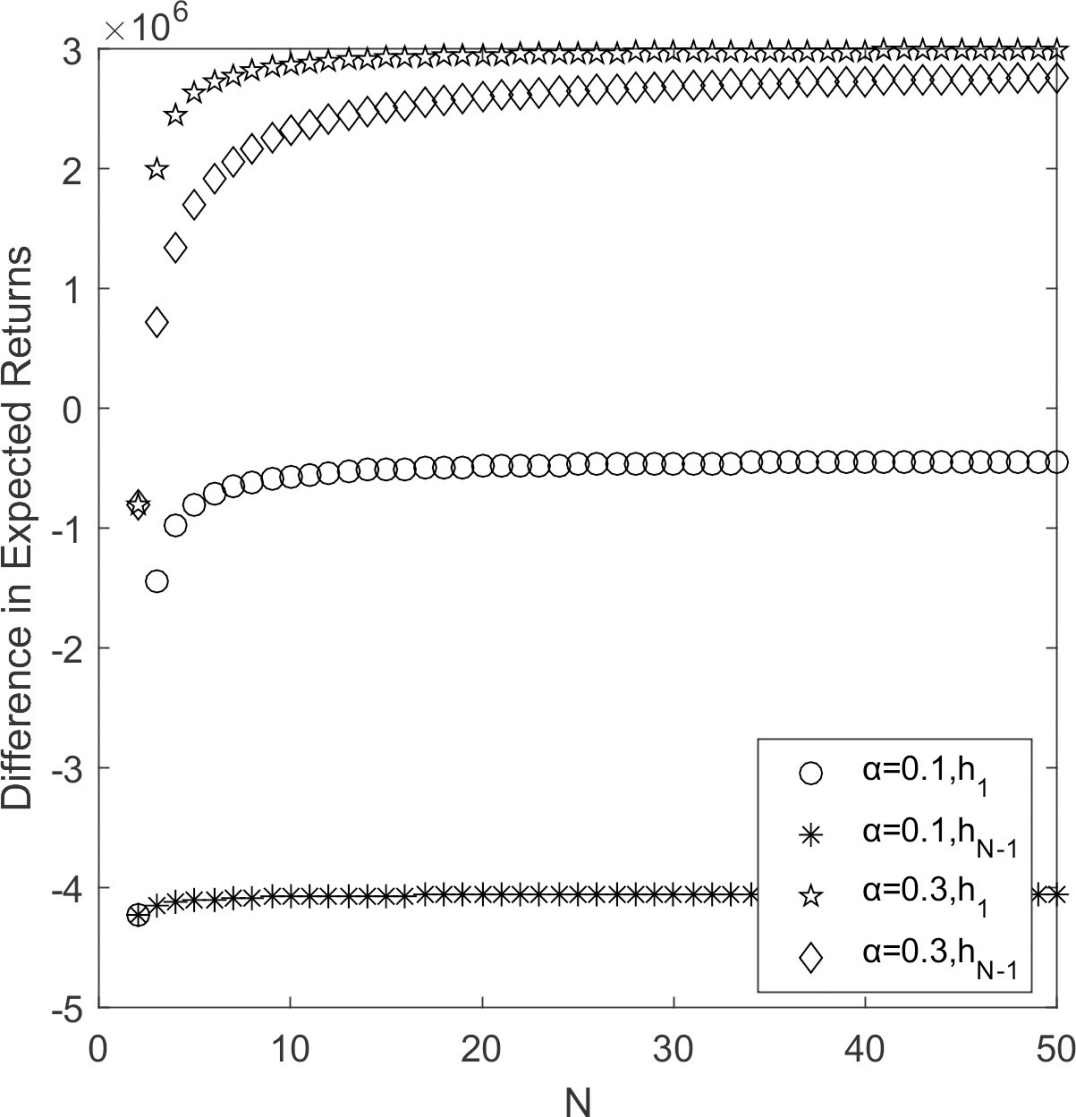
*h*_1_/*h*_*N*-1_~*α* change chart.

In Fig 2, *h*_1_ > 0and *h*_*N*-1_ > 0 when government’s subsidy coefficient for low-carbon suppliers becomes larger. According to Theorem 1, the supplier’s choice behavior supports strategy Y to invade strategy S and resists strategy S to invade strategy Y. The low-carbon emission strategy prevails. It indicates that changes in government’s subsidy coefficient affect the strategy choice of supplier populations. As the supplier population’s size expands, the two curves gradually stabilize and the difference in expected payoffs converges to a fixed value. When 2 ≤ *N* ≤ 10, the marginal effect of low-carbon emission strategy increases significantly; when *N* ≥ 10, the marginal effect of low-carbon emission strategy decreases and gradually stabilizes at a fixed value. Therefore, it is necessary for government to subsidize the cost of the supplier groups that adopts low-carbon emission strategy in conjunction with actual situation.

#### 5.1.3 Impact of government’s incentives and penalties coefficient

The evolutionary process of supplier populations when government’s rewards and punishments coefficient changes are discussed with other parameters held constant.

**Fig 3:**
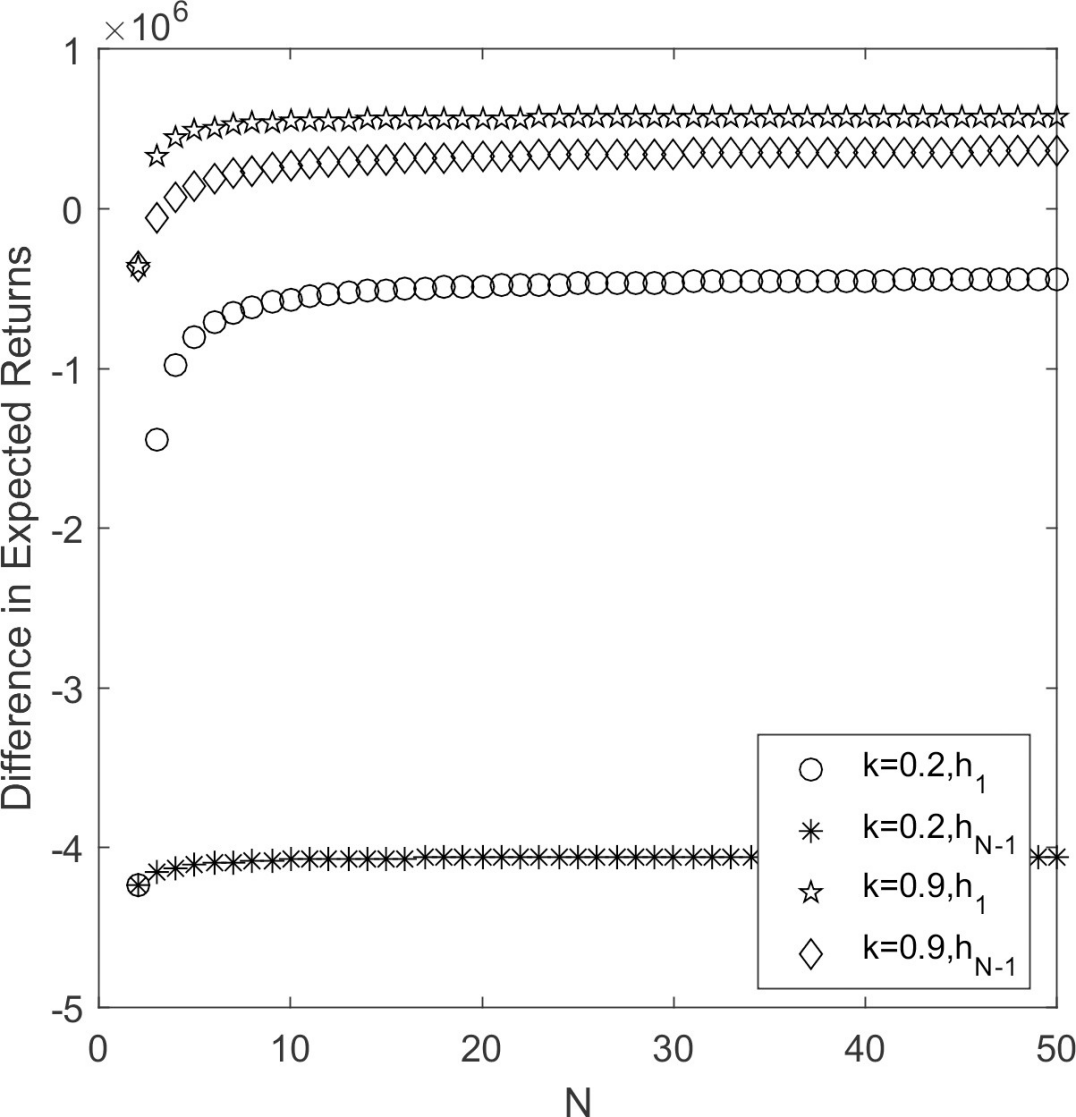
*h*_1_/*h*_*N*-1_~*k* change chart.

In Fig 3, *h*_1_ < 0and *h*_*N*-1_ < 0 when *k* = 0.2. According to Theorem 1, strategy S prevails. At *k* = 0.9, *h*_1_ > 0 and *h*_*N*-1_ < 0 when 2 ≤ *N* <7. supplier’s choice behavior tends to change, the hybrid strategy become an evolutionary stable strategy, and the marginal effect of low-carbon emission strategy has a significant upward trend. When *N* ≥ 7, *h*_1_ > 0, *h*_*N*-1_ > 0, strategy Y always become an evolutionary stable strategy with decreasing and gradually stable marginal effects at a fixed value. It indicates that government’s reward and punishment coefficient influence the strategic choice of suppliers when it becomes stronger. The difference between two curves becomes significantly smaller and finally stabilizes at a fixed value of *k* = 0.9. It indicates that as the population’s size of supplier increases, the stronger the government rewards and punishments for suppliers, the more the supplier groups tends to choose low carbon emission strategy.

#### 5.1.4 Influence of supplier’s reputation

The evolutionary process of supplier populations when the supplier’s reputation changes are discussed with other parameters held constant.

**Fig 4:**
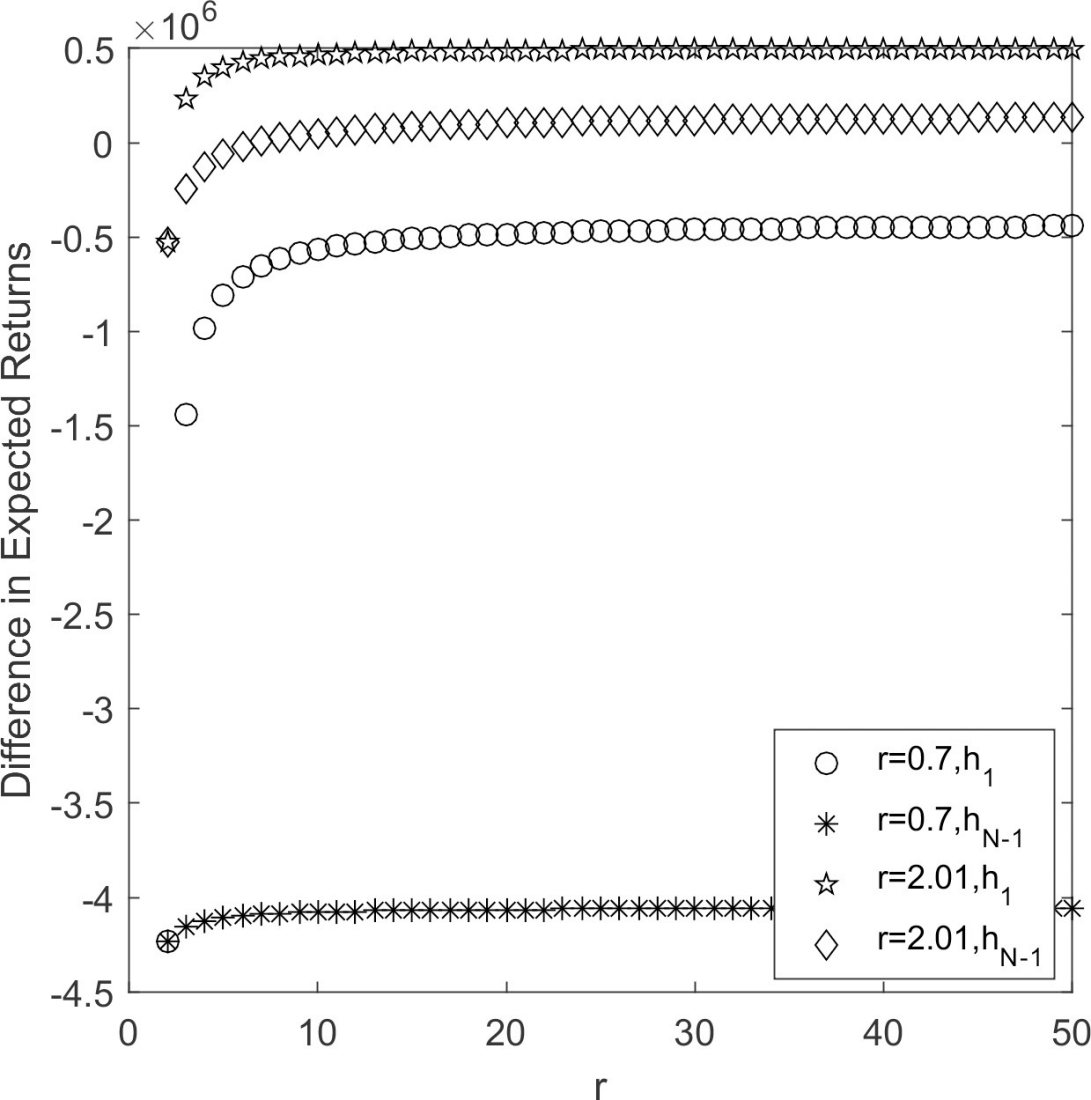
*h*_1_/*h*_*N*-1_~*r* change chart.

In Fig 4, high-carbon emission strategy becomes evolutionary stable strategy when supplier’s reputation is small. The reputation value becomes larger, and the hybrid strategy become evolutionary stable strategy when 2 ≤ *N* ≤ 10. The low-carbon emission strategy become evolutionary stable strategy when *N* > 10. The reputation of the supplier affects its strategy choice. The greater the influence of reputation, the more it tends to choose the low-carbon emission strategy. As the populations size of supplier increases, the greater the reputation, the more likely the supplier groups chooses low-carbon emission strategy.

### 5.2 Weak selection

#### 5.2.1 Effect of initial willingness of suppliers

The evolutionary process of supplier populations when initial willingness of suppliers changes are discussed with other parameters held constant.

**Fig 5:**
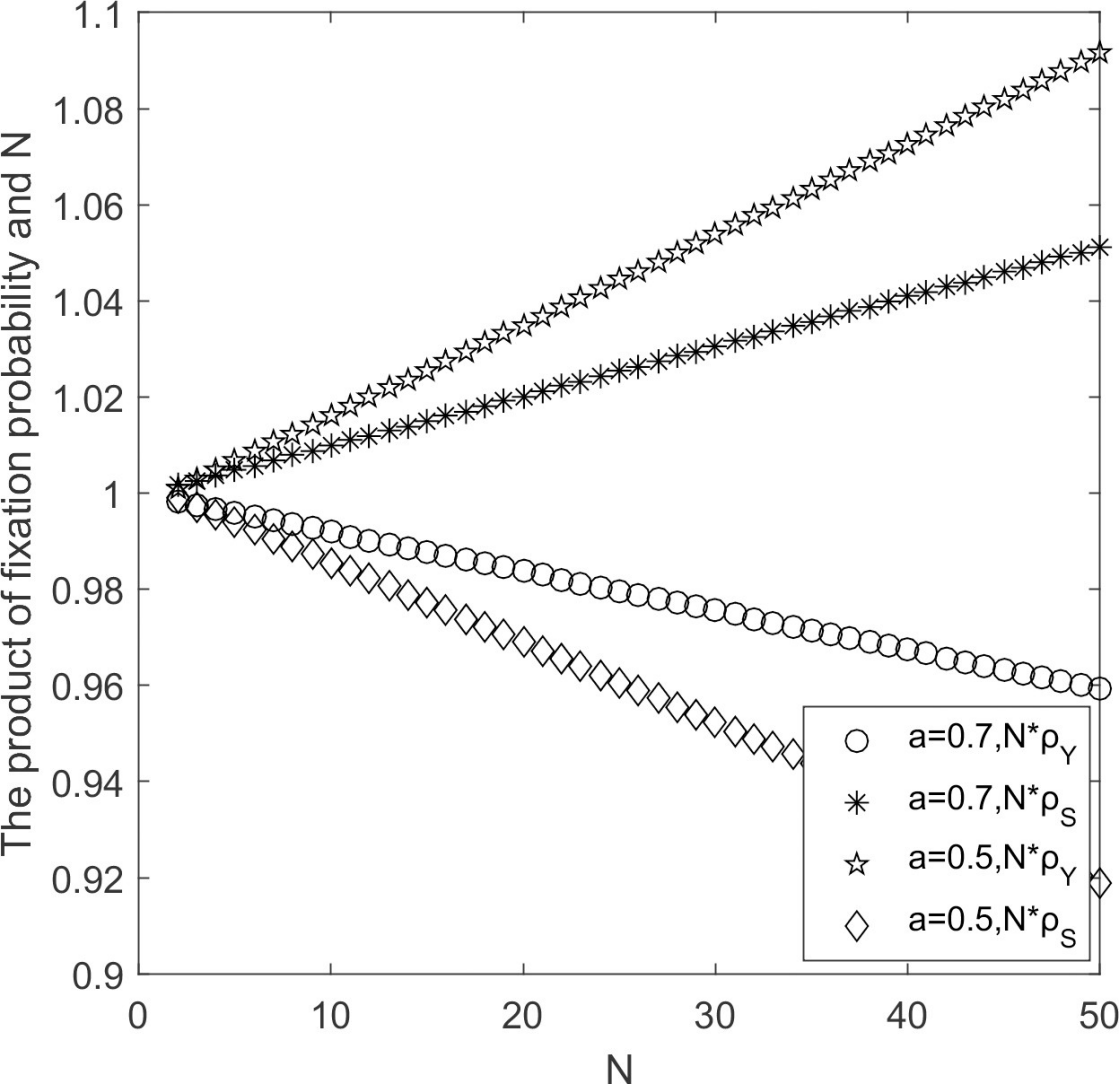
N*ρ_*Y*_/N*ρ_*S*_ ~ *a* change chart.

From Fig 5, in the weak selection situation, with larger initial willingness, ρ_*Y*_ < 1/*N* and ρ_*S*_ < 1/*N*, the high-carbon emission strategy prevails by choosing behaviorally supportive strategy S to invade strategy Y according to Theorem 2. When supplier’s initial willingness is small, ρ_*Y*_ < 1/*N* and ρ_*S*_ < 1/*N*, choose behaviorally supportive strategy Y to invade strategy S. For *N* ≥ 2 suppliers, the larger the initial willingness, the smaller the expected payoffs obtained, and strategy S is more likely to invade strategy Y. When government’s subsidy to suppliers when they adopt low-carbon emission strategy is small or absent, suppliers who choose high-carbon emission strategy obtain higher expected payoffs, and suppliers who choose high-carbon emission strategy occupy the whole populations.

#### 5.2.2 Effect of government’s cost subsidy coefficient

The evolutionary process of supplier populations when government’s cost subsidy coefficient to low-carbon suppliers change and other parameters held constant.

**Fig 6:**
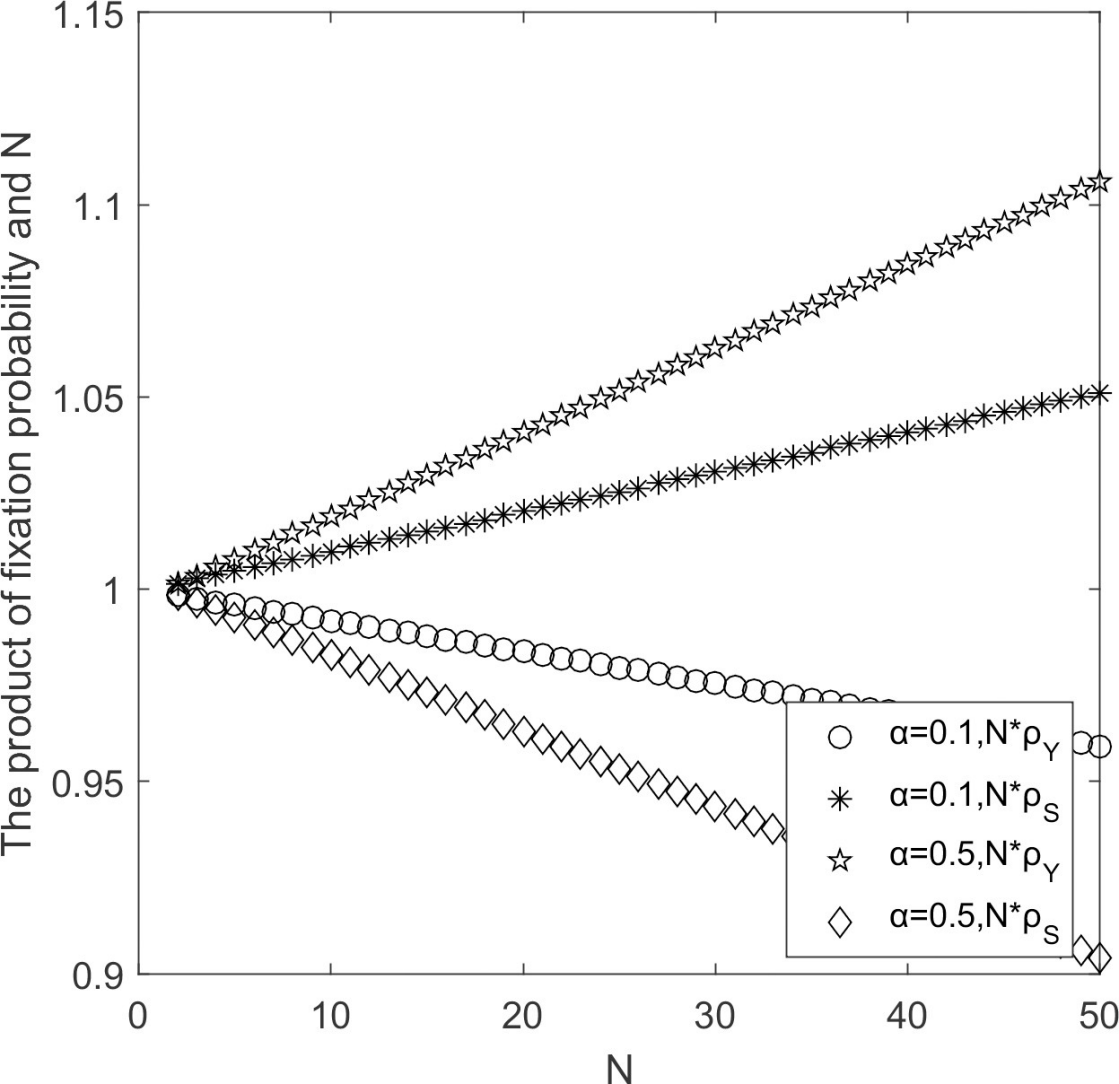
N*ρ_*Y*_/N*ρ_*S*_ ~αchange chart.

In Fig 6, when government’s cost subsidy coefficient for low-carbon suppliers is small, *ρ*_*Y*_ < 1/*N* and *ρ*_*S*_ < 1/*N*, supplier’s choice behavior support strategy S invades strategy Y and strategy S prevails. When government’s cost subsidy coefficient for low-carbon suppliers is larger, *ρ*_*Y*_ < 1/*N* and ρ_*S*_ < 1/*N*, supplier’s choice behavior support strategy Y invades strategy S and strategy Y prevails. It indicates that government’s cost subsidy is larger and the low-carbon emission strategy becomes an evolutionary stable strategy. As the government’s cost subsidy becomes larger, the trend of curve for low-carbon emission strategy changes from decreasing to increasing, while the curve for high-carbon emission strategy is reversed. With larger cost subsidies, the marginal effect of the low-carbon emission strategy changes from increasing to decreasing, and the marginal effect of the high-carbon emission strategy change from increasing to decreasing. Therefore, it is necessary for government to subsidize the cost of low-carbon suppliers.

#### 5.2.3 Influence of government’s reward and punishment intensity

The evolutionary process of supplier populations when government’s rewards and punishments coefficient to suppliers are varied are discussed with other parameters held constant.

**Fig 7:**
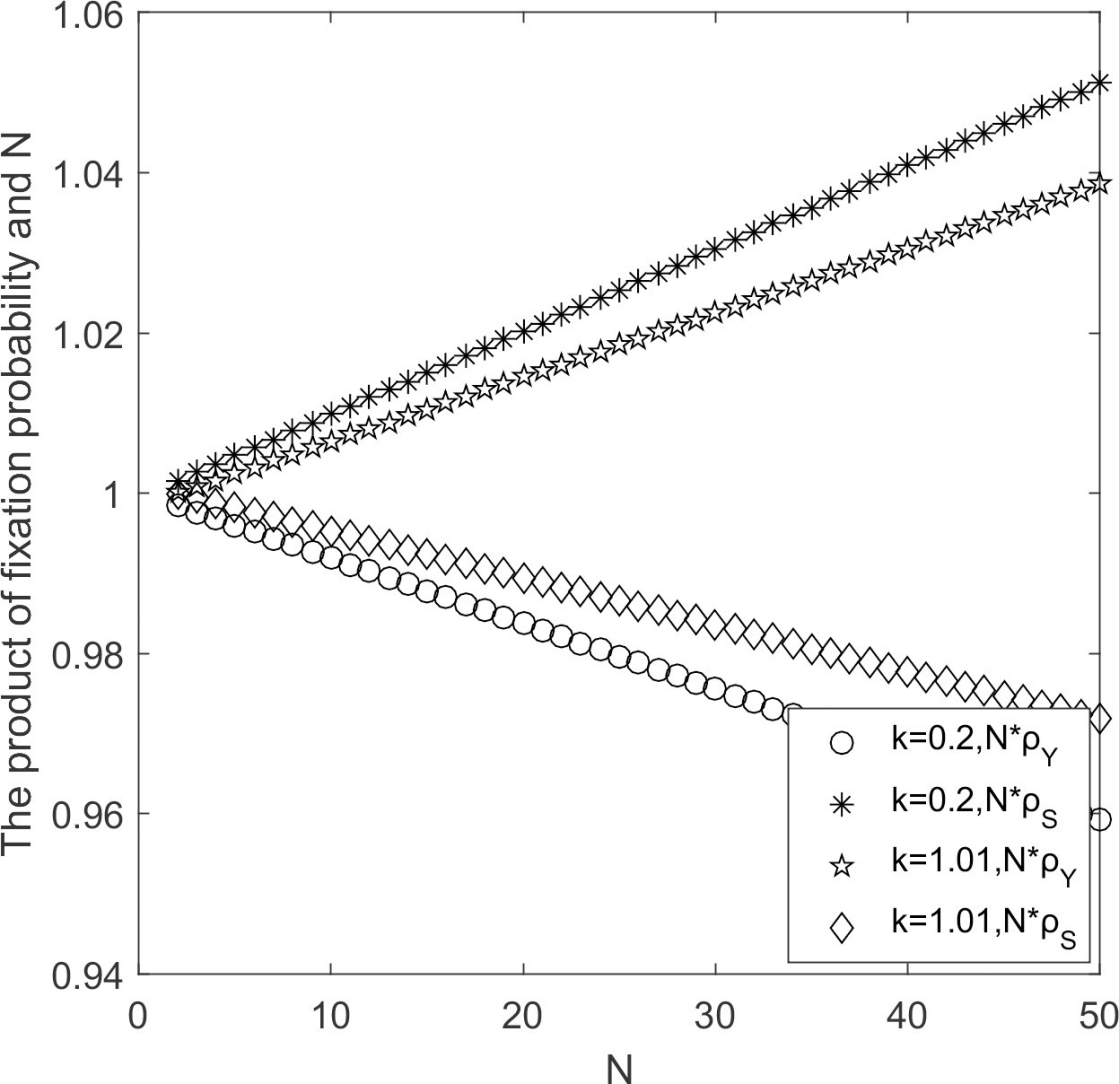
N*ρ_*Y*_/N*ρ_*S*_ ~ *k* change chart.

In Fig 7, when government’s rewards and punishments coefficient for supplier’s strategy selection are small, ρ_*Y*_ < 1/*N* and ρ_*S*_ < 1/*N*, according to Theorem 2, supplier’s choice behavior is in favor of high-carbon emission strategy to invade low-carbon emission strategy. When rewards and punishments coefficient become stronger, ρ_*Y*_ < 1/*N* and ρ_*S*_ < 1/*N*, supplier’s choice behavior supports strategy Y to invade strategy S and resists strategy S to invade strategy Y. It suggests that effective government incentives for suppliers influence their strategy choice. The greater the incentives and penalties, the more the low-carbon emission strategy prevails. The trend of low-carbon emission strategy curve changes from decreasing to increasing, and the marginal effect changes from increasing to decreasing; the curve of high-carbon emission strategy changes from increasing to decreasing, and the marginal effect changes from increasing to decreasing.

#### 5.2.4 Effect of supplier’s reputation

The evolutionary process of supplier populations when supplier’s reputation changes are discussed with other parameters held constant.

**Fig 8:**
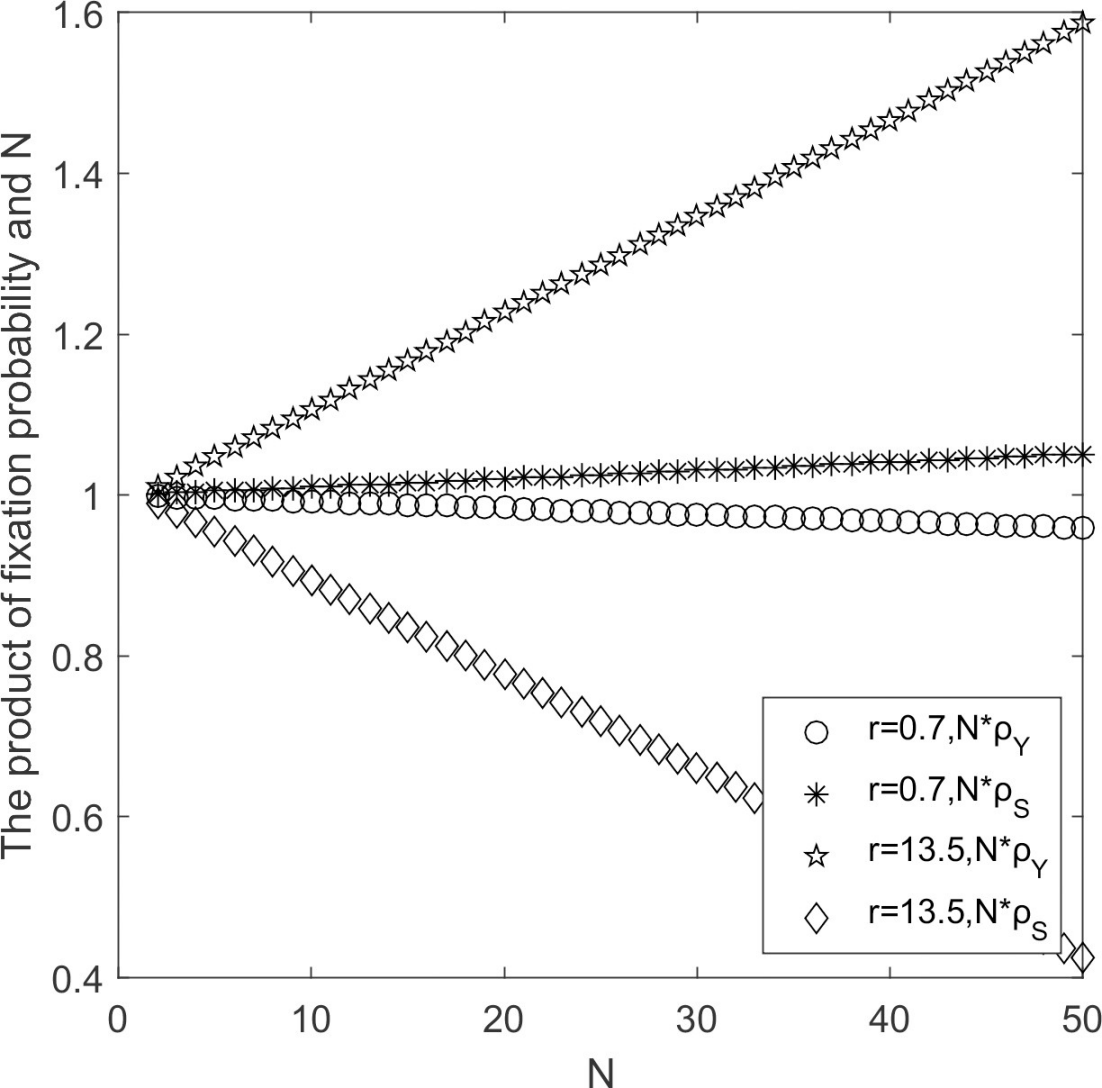
N*ρ_*Y*_/N*ρ_*S*_~ *r* change chart.

In Fig 8, when supplier’s reputation influence is small, ρ_*Y*_ < 1/*N* and ρ_*S*_ < 1/*N*, by Theorem 2, supplier’s choice behavior support strategy S to invade strategy Y and strategy S prevails. When supplier’s reputation influence increases, ρ_*Y*_ < 1/*N* and ρ_*S*_ < 1/*N*, supplier’s choice behavior support strategy Y invades strategy S and strategy Y prevails. The greater the supplier’s reputation influence, the more it tends to choose low-carbon emission strategy. As the supplier’s population’s size increases, the possibility of low-carbon emission strategy becomes an evolutionary stable strategy increases greatly.

### 5.3 Unconditional average fixation time

Suppose there are *i* suppliers choosing strategy Y within populations at the beginning, and the number of individuals choosing strategy Y within populations may be 0 or N as time evolves. Let N = 20, *ω* = 0.001, and discuss the evolution of supplier populations as initial willingness of suppliers and government’s cost subsidy coefficient change, with other parameters held constant.

**Fig 9:**
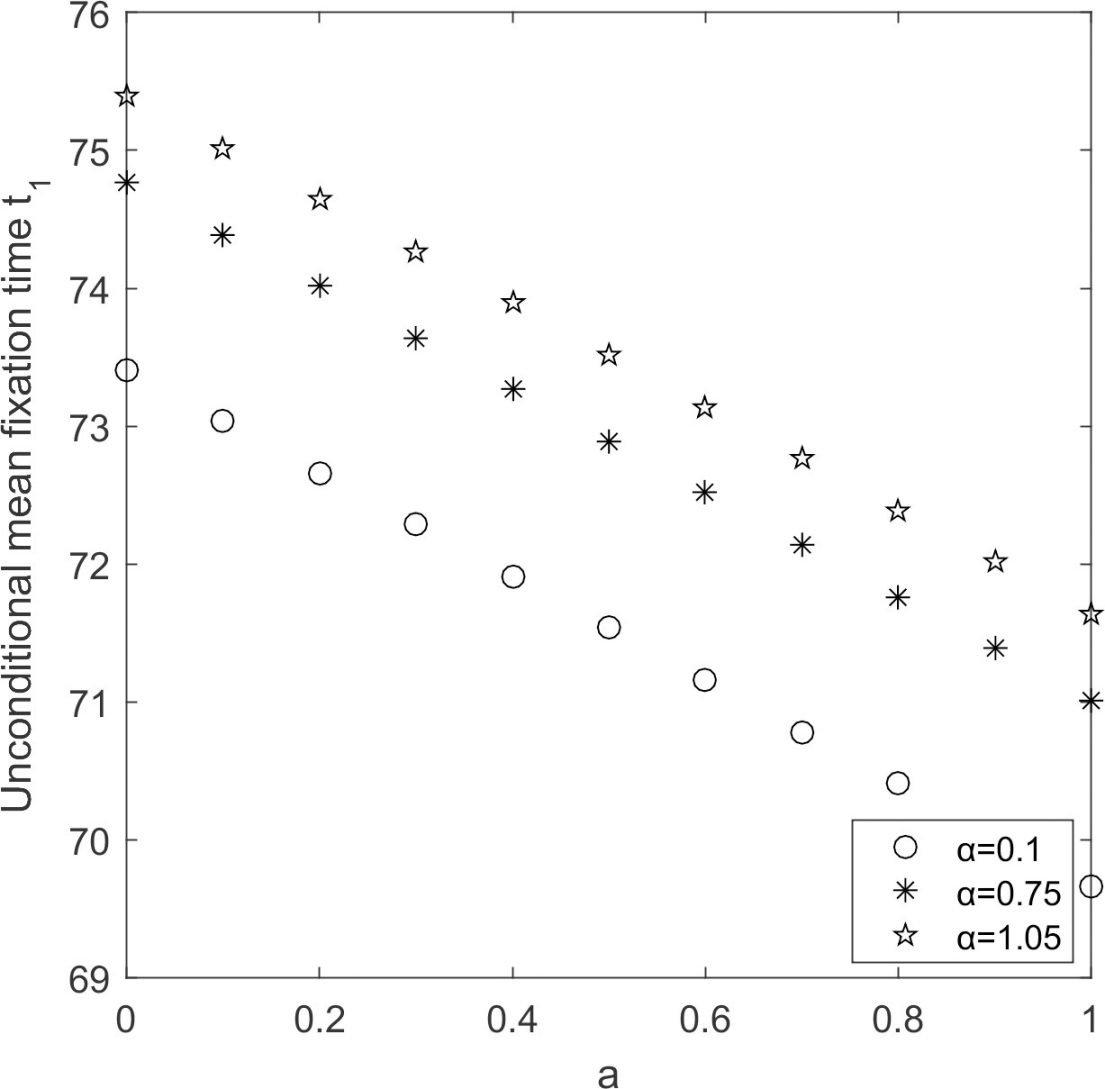
*t*_1_~αchange chart.

In Fig 9, unconditional average fixation time decreases with the increasing in the initial willingness of suppliers and increases with the increasing in government’s cost subsidy coefficient. As obtained from Figs 2 and 6, the larger the government’s cost subsidy coefficient, the more dominant the low-carbon emission strategy is. Combined with Fig 9, the larger the initial willingness and the larger the cost subsidy coefficient, the shorter the time it takes for low-carbon emission strategy to fixate and the faster the supplier groups will shift to low-carbon production. Meanwhile, as the initial willingness increases and the cost subsidy coefficient increases, the number of suppliers choose high-carbon emission strategy decreases and the time for supplier groups to evolve to all suppliers choose high-carbon emission strategy to fixate increases.

Let N = 20, *ω* = 0.001, under the condition that other parameters remain unchanged, discuss the evolution process of the supplier groups when the initial willingness of the supplier groups and the government’s reward and punishment coefficient change.

**Fig 10:**
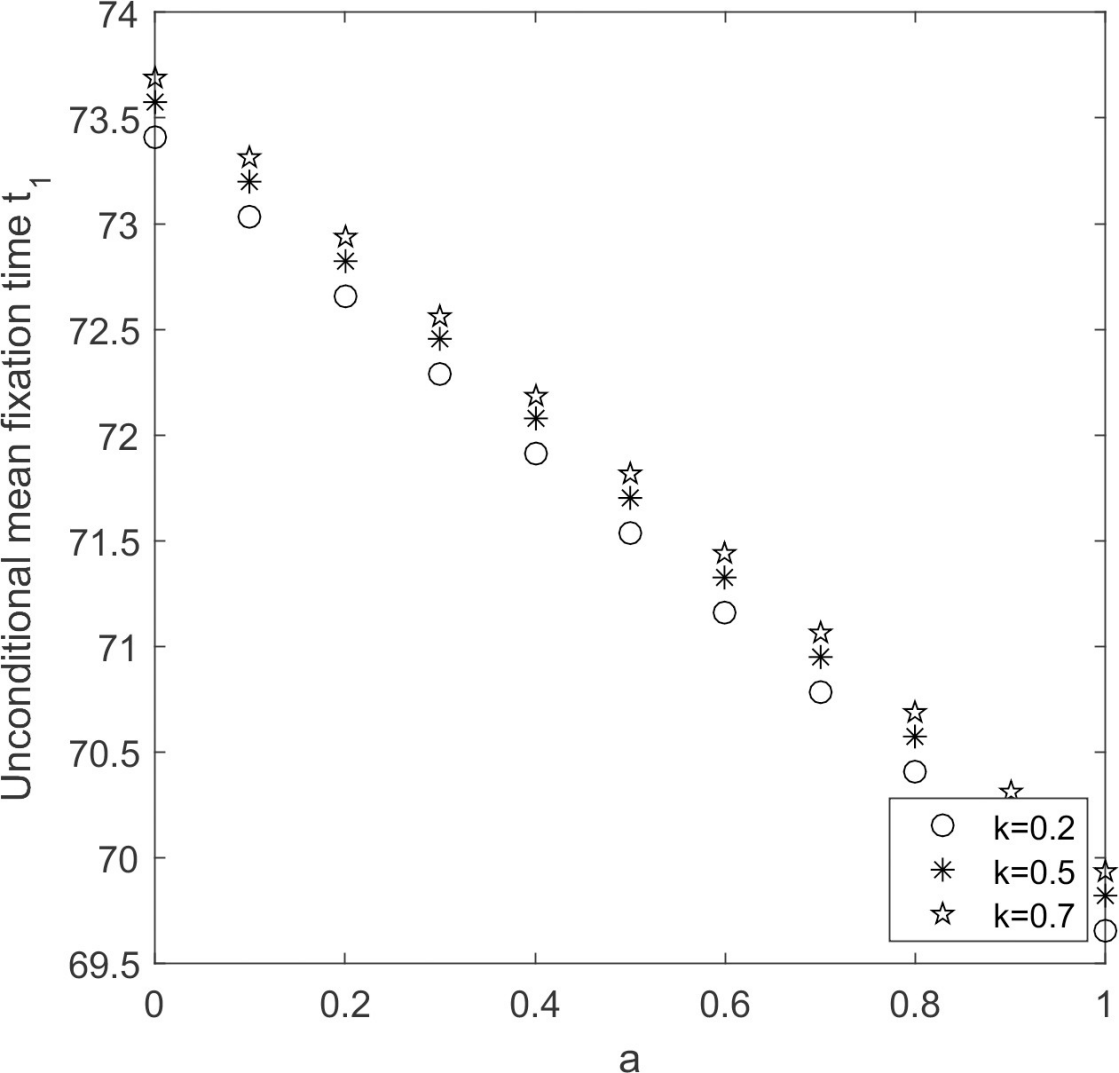
*t*_1_~ *k* change chart.

From Fig 10, it can be seen that unconditional average fixation time decreases with the increasing of supplier’s initial willingness to choose low-carbon emission strategy and increases with the increasing of government’s incentives and penalties. From Figs 3 and 7, it is obtained that the greater the incentive and punishment coefficient, the more dominant the low-carbon emission strategy. Combined with Fig 10, the larger the supplier’s initial willingness to choose low-carbon emission strategy, the greater the intensity of rewards and penalties coefficient, the shorter the time needed for low-carbon emission strategy to fixate, and the faster the supplier groups shifts to low-carbon production. As supplier’s initial willingness increases and government’s rewards and punishments increase, the time for supplier individuals choose high-carbon emission strategy to occupy supplier group increases.

Let N = 20, *ω* = 0.001 discuss the evolutionary process of supplier groups when initial willingness of suppliers and supplier’s reputation change, with other parameters held constant.

**Fig 11:**
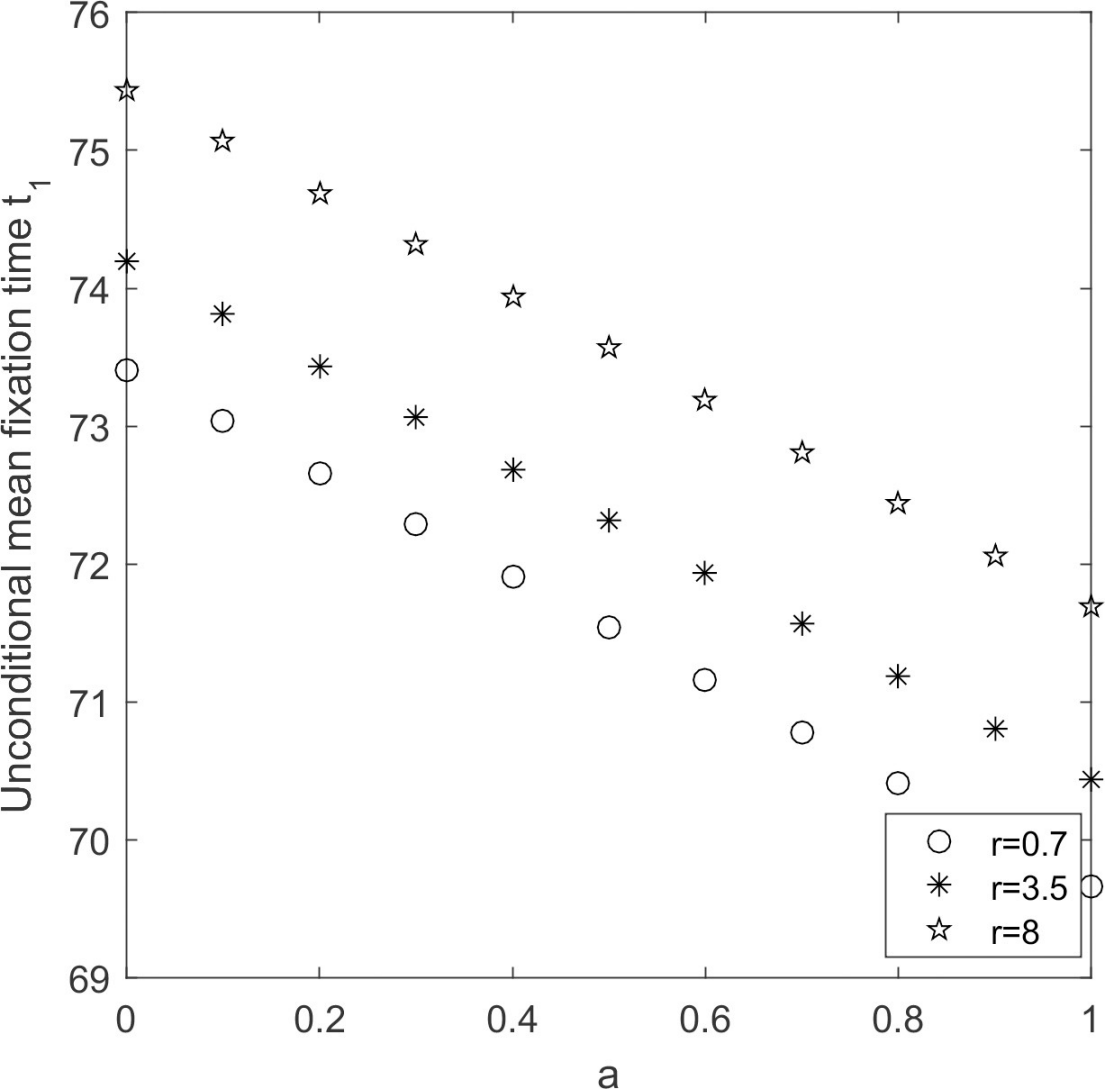
*t*_1_~*r* change chart.

In Fig 11, unconditional average fixation time decreases with increasing initial willingness and increases with increasing supplier’s reputation. From Figs 4 and 8, the greater the supplier’s reputation value, the greater the probability that low-carbon emission strategy fixate Combined with Fig 11, the greater the initial willingness of suppliers to choose low-carbon emission strategy and the greater the supplier’s reputation value, the shorter the time it takes for low-carbon emission strategy to fixate and the faster the supplier groups shifts to low-carbon production.

### 5.4 Conditional average fixation time

The evolutionary process of supplier populations is discussed with supplier’s selection intensity varying.

**Fig 12:**
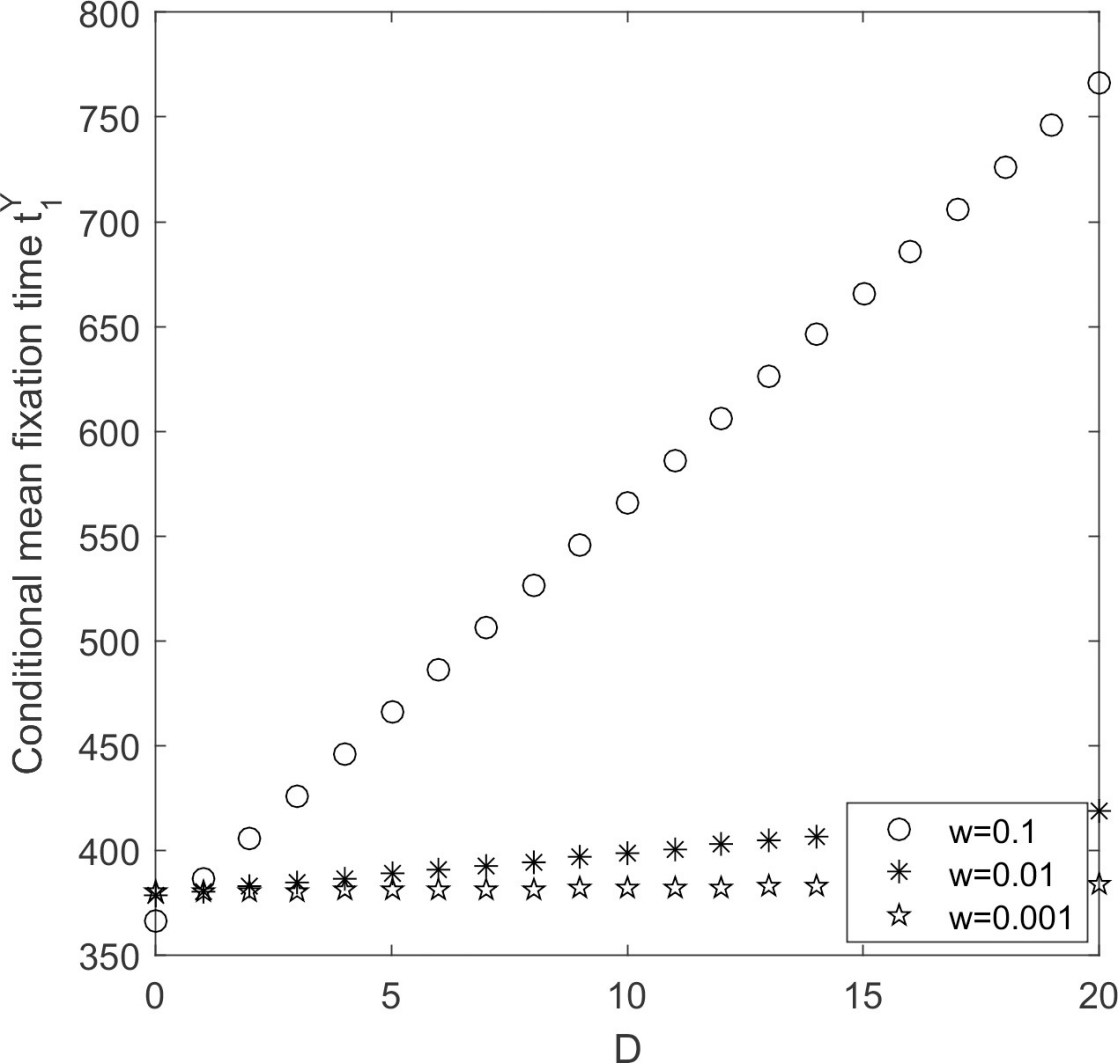
$t_{1}^{Y}$ ~ ω change chart.

In Fig 12, when industry recognition is at a fixed value, conditional average fixation time increases with the increasing of selection intensity. When selection intensity is constant, conditional average fixation time increases with the declining in industry recognition. The government must take various measures to reduce the risks of suppliers when they choose low-carbon emission strategy, and strive to increase their motivation to choose low-carbon emission strategy.

Under the condition that other parameters remain unchanged, discuss the evolution process of supplier groups when supplier’s populations changes.

**Fig 13:**
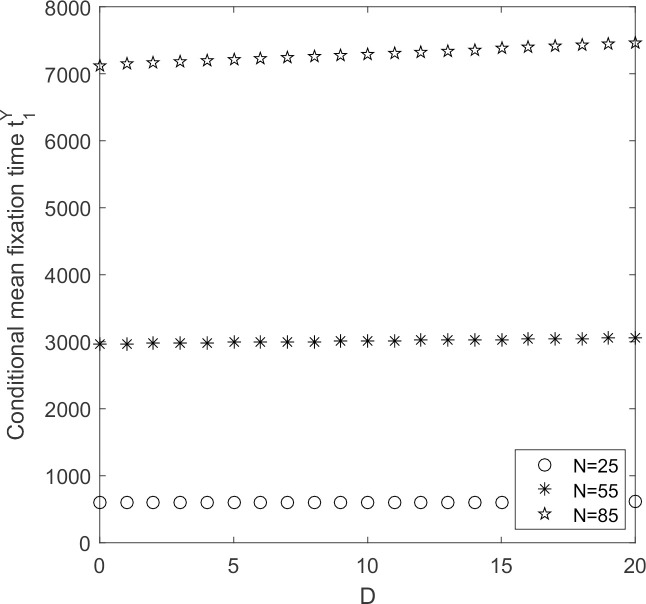
$t_{1}^{Y}$~N change chart.

In Fig 13, the larger the supplier population’s size, the longer the time it takes for low-carbon emission strategy to take hold as industry acceptance continues to decline. There are a large variety and different sizes of suppliers in construction industry. Only by changing from traditional to low-carbon production models for various types of suppliers as soon as possible can raise the recognition of the construction industry as a whole as soon as possible.

## 6 Conclusions and implications

### 6.1 Conclusions

To achieve low-carbon production in construction industry requires the joint efforts of government and enterprises. This paper studies the evolutionary trajectory of the construction engineering supplier group’s transition to low-carbon production under government’s carbon regulatory environment from the perspective of the engineering supply chain supplier groups. A stochastic evolutionary game model of supplier choosing low-carbon emission strategy is established based on Moran process. This model mainly solves the following problems: what factors affect the supplier’s choice of low-carbon strategy? How to turn suppliers to low carbon production mode as soon as possible? Some suggestions for government regulation based on numerical simulation. The research results are as follows.

- The supplier’s strategy selection behavior is related to supplier’s initial willingness, government’s cost subsidy coefficient for low-carbon suppliers, government’s rewards and punishments, and supplier’s reputation.
- When supplier group’s size are fixed and the initial willingness of suppliers to choose low-carbon emission strategy are large, the greater the cost subsidy coefficient is, the greater the reward and punishment coefficient is, and the greater the supplier’s reputation is, the more stable the supplier groups evolutionary strategy are toward low-carbon production strategy, and the faster the supplier groups shifts to the low-carbon production strategy. As supplier group’s size increases, the marginal effects of cost subsidies, incentives and punishments for risk-preferred supplier groups diminish.
- The larger the initial willingness, the greater the cost subsidy coefficient, the greater the rewards and punishments are, the greater the credibility is, the shorter the time needed to fixate in low-carbon emission strategy, and the faster the supplier group turns to low-carbon production. As supplier’s initial willingness increases, government’s subsidy coefficient increases, the number of suppliers choosing high-carbon strategy decreases, and the time for supplier group to evolve to adopt all the high-carbon strategies increases. It should also be noted that the marginal effect of subsidy strength is related to supplier population’s size and risk attitude; when supplier population’s size is small, the marginal utility and risk attitude are unrelated; when the supplier population’s size is large, the marginal effect and risk attitude are related. In the strong selection situation, suppliers are risk-averse, and the marginal effect has a clear upward trend within a certain populations range, and decreases and gradually stabilizes at a fixed value as the population’s size increases. In the situation of weak selection, suppliers are risk-preferred, and the marginal effect changes from increasing to decreasing as the subsidy increases, whether it is a low-carbon emission strategy or a high-carbon emission strategy.
- When industry recognition is at a fixed value, conditional average fixation time increases with the increasing of selection intensity. When selection intensity is constant, conditional mean fixation time increases with the declining in industry recognition. The larger the supplier population’s size, the longer the time it takes for low-carbon emission strategy to take hold as industry acceptance continues to decline.

### 6.2 Implications

Based on the above discussion, the government can guide supplier groups to transformation into low-carbon production by following means.

- Establish carbon emissions standards and related management regulations in construction industry as soon as possible to guide industry enterprises to transition to low-carbon production. Due to influenced by various factors, without the guidance of management regulations, the stochastic evolution of engineering supplier groups may result in low-carbon emission strategy or high-carbon emission strategy, so determining carbon emissions standards and setting relevant management regulations are the necessary environmental conditions to guide engineering suppliers to transition to low-carbon production.
- Appropriate cost subsidies for suppliers of low-carbon production. When suppliers choose low-carbon production, improving production processes, using clean energy, and applying carbon capture technologies require significant additional costs, and the emission reduction cycle is long. Even if the initial willingness of suppliers to choose low-carbon emission strategy is large, if the government doesn’t subsidize suppliers or the subsidy is small, high-carbon emission strategy will become evolutionary stable strategy. Therefore, government can subsidize the cost of low-carbon production suppliers by appropriately reducing taxes and other means to guide supplier groups to change to low-carbon production mode.
  The institutional path that the government should take is: firstly, the government should increase the publicity of environmental protection and entrepreneurship to encourage suppliers to develop innovation and improve the risk appetite of enterprises; then, a differentiated subsidy policy should be implemented for suppliers in the construction industry. Due to the large size and many categories of the construction industry supplier groups, they provide different products and services, have different ability to take risks, and have different risk attitudes. Therefore, for a smaller number of risk-averse suppliers, such as special material producers, new material producers, special equipment producers, construction companies with special processes or technologies, etc., the main approach should be taken to cost subsidies, and the subsidy should be appropriately increased; for suppliers with homogeneous products or services and a large number of suppliers, due to the diminishing marginal effect of cost subsidies, so subsidy cost should be appropriately controlled, the strength of policy publicity should be enhanced.
- Set up corresponding reward and punishment mechanisms to reward suppliers who choose low-carbon emission strategy and penalize suppliers who choose high-carbon emission strategy. Regardless of whether suppliers make decisions based entirely on expected payoffs or make decisions based on a combination of policy orientation, risk preferences and other factors, the larger the government’s reward and punishment coefficients for suppliers when supplier population’s size is certain, the greater the probability of low-carbon emission strategy becoming an evolutionary stable strategy. Moreover, when supplier’s initial willingness to choose low-carbon strategy is larger, the greater the government’s reward and punishment for suppliers, the faster the supplier populations shifts to low-carbon production transition. However, it should also be noted that similar to cost subsidies, the marginal effect of the reward and punishment mechanism is also related to supplier’s populations size and risk attitude of suppliers, and the marginal effect of reward and punishment mechanism decreases when the number of suppliers is large and risk preferences are high, so for suppliers with homogeneous products or services and a large number of suppliers, the intensity of reward and punishment can be controlled appropriately, while the intensity of policy promotion can be increased.
- Build a carbon emissions information disclosure platform and disclose government’s inspection results to enhance the credibility value of suppliers. The high-carbon emission strategy prevails when the supplier population’s size is certain and reputation is small. When supplier’s reputation value is larger, the low-carbon emission strategy prevails. With the expansion of population’s size, when the supplier’s reputation value is larger, the low-carbon emission strategy eventually becomes an evolutionary stable strategy and the recognition of the whole construction industry rises. When supplier’s initial willingness to choose low-carbon emission strategy is larger, the shorter the time for low-carbon strategy to fixate as supplier’s reputation increases, the faster the transformation of supplier populations to low-carbon production. Therefore, a carbon emissions information disclosure platform should be built to form a system of public inspection results and applying such inspection results to enhance the value of supplier’s credibility, for example, a green credit assessment and evaluation mechanism based on carbon emissions inspection results can be established to give credit priority or preferences to enterprises with better carbon emissions credibility; in bidding for engineering materials, bidding for engineering equipment, and bidding for engineering construction, carbon emissions evaluation criteria should be added to enhance the market competitiveness of enterprises with better carbon emission reputation, etc.

## Supporting information

S1 Fig (RAR)

## References

[1] Agency XN. Xi Jinping delivers important speech at 75th UN general assembly general debate http://www.gov.cn/xinwen/2020-09/22/content_5546168.htm2020

[2] Ma XM, Cai Y. China low carbon finance development report. Peking University Press; 2014.

[3] Jin HY, Li Y. CBMF held a pep talk to promote the industry’s carbon peak and carbon neutral action. China Building Materials. 2021(02):24–25.

[4] Federation CBM. Proposal for promoting carbon peak and carbon neutral action in the building materials industry. China Building Materials. 2021(02):21–23.

[5] Jin HY, Han Y, Li Y. Carbon peak, carbon neutral—big country ambition building materials industry must play the role China Building Materials. 2021(02):26–33.

[6] Fang H, Cao ZL, Feng ZH, Chen SX. Carbon emission accounting method and empirical research of highway construction period based on full life cycle theory. Highway Engineering. 2021;46(01):92–97+124.

[7] Hu S, Zhang Y, Yan D, Guo SY, Liu Y, Jiang Y. Definition and modeling of energy consumption and carbon emissions in China’s building sector. Building Science. 2020(36(S02)):288–297.

[8] Gao Y, Fang L, Xue GX. Multi objective optimization algorithm for carbon emission evaluation function of building life cycle. Computer Simulation. 2021;38(02):240–243+405.

[9] Li J, Liu Y. The carbon emission accounting model based on building lifecycle. Journal of Engineering Management. 2015;29(04):12–16.

[10] Sun JY, Di YQ. Research on low carbon emission regulatory mechanism in China’s construction sector. Construction Science and Technology. 2015(02):64–65.

[11] Yang T, Xue S. Research on endogenous mechanism of green transformation driven by construction enterprises from the perspective of technology and institution interaction. Modernization of Management. 2020;40(3):16–19.

[12] Fu FF. A study on carbon emission measurement of steel construction process based on discrete simulation. Journal of Engineering Management. 2018;32(01):98–103.

[13] Yin S, Li BZ, Xing ZY. The governance mechanism of the building material industry (BMI) in transformation to green BMI: The perspective of green building. The Scienceof the Total Environment. 2019;677:19–33. doi: 10.1016/j.scitotenv.2019.04.317 31051380

[14] Jabbour ABLdS Júnior SAV, Jabbour CJC Filho WL, Campos LS Castro RD. Toward greener supply chains: is there a role for the new ISO 50001 approach to energy and carbon management? Energy Efficiency. 2016;10(3):777–785.

[15] Monasterolo I, Battiston S, Janetos AC, Zheng Z. Vulnerable yet relevant: the two dimensions of climate-related financial disclosure Climatic Change. 2017;145(3–4):495–507.

[16] Nishitani K, Kokubu K, Kajiwara T. Does low-carbon supply chain management reduce greenhouse gas emissions more effectively than existing environmental initiatives? An empirical analysis of Japanese manufacturing firms. Journal of Management Control. 2015;27(1):33–60.

[17] He QH, Wang G. The developments and trends of green and low-carbon building study based on scientometric map. Forum on Science and Technology in China. 2015(10):136–141.

[18] Wang YW, Xue XL. The application of supply chain management in construction industry. China Civil Engineering Journal. 2004;37(9):86–91.

[19] Wang HC, He BZ. Research on the application of green supply chain management in China’s construction industry. Construction Economy. 2010(6):31–33.

[20] Robati M, Daly D, Kokogiannakis G. A method of uncertainty analysis for whole-life embodied carbon emissions (CO2-e) of building materials of a net-zero energy building in Australia. Journal of Cleaner Production. 2019;225:541–553.

[21] Choi J, Lee MG, Oh HS, Bae SG, An JH, Yun DY, et al. Multi-objective green design model to mitigate environmental impact of construction of mega columns for super-tall buildings. Science of the Total Environment. 2019;674:580–591. doi: 10.1016/j.scitotenv.2019.04.152 31022547

[22] Liu Z, Zhu Y. Sustainability performance evaluation of construction corporations. China Civil Engineering Journal. 2009;42(7):131–134.

[23] Du S, Hu L, Wang L. Low-carbon supply policies and supply chain performance with carbon concerned demand. Annals of Operations Research. 2015;255(1–2):569–590.

[24] Zhang WY, Liu CG, Li L, Jiao JX. Allocation of the carbon emission abatement target in a two-echelon supply chain. Chinese Journal of Management Science. 2021;29(09):90–101.

[25] Lin H, Ma C, Sun Q, Li D. Research on carbon reduction optimization strategy and coordination mechanism in consideration of corporate social responsibility. Operations Research and Management Science. 2021;30(01):29–35.

[26] Xu L, Wang C, Li H. Decision and coordination of low-carbon supply chain considering technological spillover and environmental awareness. Scientific Reports. 2017;7(1):3107. doi: 10.1038/s41598-017-03270-2 28596560 PMC5465093

[27] Zheng J, Zhou Y. Will Enterprises Choose Low-carbon Production Technology? Evolutionary Game Model Considering Consumers’ Environmental Protection Moral and Market Clearing. IOP Conference Series: Materials Science and Engineering. 2020;790(1). doi: 10.1088/1757-899X/790/1/012126

[28] Zhang LR, Peng B, Cheng CQ. Research on government subsidy strategy of low-carbon supply chain based on block-chain technology. Chinese Journal of Management Science. 2021:1–13. (Forthcoming)

[29] Zhang LH, Kong YW, Wang JY. Equilibrium strategies of enterprise’s two-stage production with strategic consumer’s low-carbon preference. Computer Integrated Manufacturing Systems. 2020;26(11):3167–3176.

[30] Feng ZW, Liu DW. Emission reduction decision in autonomous reduction low-carbon supply chain with loss aversion. Computer Integrated Manufacturing Systems. 2021:1–21. (Forthcoming)

[31] Song H, Zhu J, Dai Y. Sharing mechanism of surplus carbon emission quota in supply chain enterprises under imperfect carbon trading market. Computer Integrated Manufacturing Systems. 2016;22(09):2217–2226.

[32] Jiang JX, He XH, Hu WF. Research on emission reduction strategies of three-tier supply chain considering carbon subsidies and corporate social responsibility. Systems Engineering. 2021:1–19. (Forthcoming)

[33] Zhang LH, Zhang GW, Zhang R. Equilibrium contracts strategies of low carbon supply chain and high carbon supply chain. Computer Integrated Manufacturing Systems. 2018;24(03):763–771.

[34] Zhang LR, Yang ZF, Cheng CQ. Selection of emission reduction strategy for closed-loop supply chain under carbon cap-and-trade policy. Journal of Industrial Engineering and Engineering Management. 2021:1–9. (Forthcoming)

[35] Wei SD, Sun M. Analysis on strategy of carbon emission reduction R&D between supply chains under consumer subsidy. Computer Integrated Manufacturing Systems. 2021:1–24. (Forthcoming)

[36] Zhang LH, Song XB, Zhang GW, Lin GL. Adoption and coordination of carbon reduction technology in supply chain based on carbon tax. Computer Integrated Manufacturing Systems. 2017;23(04):883–891.

[37] Yang YX, Zhang BY, Meng LJ, Yu YN. Effect of different carbon tax policies on the supply chain network equilibrium. Computer Integrated Manufacturing Systems. 2020:1–19. (Forthcoming)

[38] Zhang KY, Li CX, Yao JM, Li JX. Research on low carbon supply chain financing decision under the purchase capital constraint of the retailer. Operations Research and Management Science. 2021;30(08):108–116.

[39] Lu JC, OuYang HX, Han L. Evolutionary mechanism of low-carbon transformation of construction enterprises under multi-agent interaction games. Journal of Beijing Institute of Technology (Social Sciences Edition). 2019;21(01):17–26.

[40] Liu XM, Sun XY, Wu SJ. Evolution of carbon emission game under dual governance system: Analysis from the perspective of initial willingness differentiation. Systems Engineering. 2019;37(03):31–47.

[41] Wu SJ, Sun XY, Yang P. Game analysis of carbon emission regulation under dual governance system. China Population, Resources and Environment. 2017;27(12):21–30.

[42] Zhang KZ, Lu JQ, Xu SS, Sun FH. Research of evolutionary game and strategy between government and enterprises in carbon emission supervision: Based on the perspective of the third party. Journal of Chongqing University (Social Science Edition). 2020;26(04):82–92.

[43] Liang W, Chen GQ, Zhang Q. A game analysis on the production of low carbon products by core enterprises in the supply chain under the supervision of carbon emission. Journal of Beijing Jiao tong University (Social Sciences Edition). 2017;16(02):137–145.

[44] Traulsen A, Claussen JC, Hauert C. Coevolutionary dynamics: from finite to infinite populations. Physical Review Letters. 2005;95(23):238701. doi: 10.1103/PhysRevLett.95.238701 16384353

[45] Taylor C, Fudenberg D, Sasaki A, Nowak MA. Evolutionary game dynamics in finite populations. Bulletin of Mathematical Biology. 2004;66(6):1621–1644. doi: 10.1016/j.bulm.2004.03.004 15522348

[46] Perc M. Phase transitions in models of human cooperation. Physics Letters A. 2016;380(36):2803–2808.

[47] Traulsen A, Shoresh N, Nowak MA. Analytical results for individual and group selection of any intensity. Bulletin of Mathematical Biology. 2008;70(5):1410–1424. doi: 10.1007/s11538-008-9305-6 18386099 PMC2574888

[48] Wang XJ, Gu CL, Zhao JH, Quan J. A review of stochastic evolution dynamics and its cooperative mechanism. Journal of Systems Science and Mathematical Sciences. 2019;39(10):1533–1552.

[49] Chai CC, Xiao TJ, Xu TT. Evolutionary dynamics of manufacturers’ production strategies based on Moran Process. Systems Engineering Theory & Practice. 2015;35(09):2262–2270.

[50] Yang FM, Wang AY, Wu J, Tang L. Evolutionary dynamics of E-commerce platform’s credit information sharing strategy. Journal of Systems Engineering. 2017;32(05):596–603.

[51] Wang XJ, He QL, Quan J, Gu CL. Evolutionary dynamics of consumers’ crowd funding strategies based on Moran process. Operations Research and Management Science. 2017;26(11):105–110.

[52] Sun SH, Zhu LL. Moran Evolutionary analysis of enterprise’s quality improvement investment decision under consumer feedback. Chinese Journal of Management Science. 2020:1–12. (Forthcoming)

[53] Wu SS, Liu DH, Wang L. Moran process stochastic evolution model of anti-terrorism de-extremalization source governance strategy. Chinese Journal of Management Science. 2021:1–12. (Forthcoming)

[54] Wu SS, Liu DH, Wang L. Stochastic evolutionary game model of Moran process for attacking strategies of decentralizing terrorist organizations. Systems Engineering Theory & Practice. 2020;40(11):2885–2896.

[55] Song MX, Liu DH. Stochastic evolutionary game model for resolution mechanism of mass events. Chinese Journal of Management Science. 2020;28(04):142–152.

[56] Wang TH, Liu DH, Wang L. Stochastic evolutionary game model of international anti-terrorism alliance under uncertain environment. Systems Engineering Theory & Practice. 2019;39(12):3139–3150.

[57] Wang TR, Guo JY, Wang HT, Huang F. Evolution dynamics of opportunistic behavior of PPP project contractors based on Moran process. Journal of Technology Economics. 2020;39(08):168–173.

[58] Nowak MA, Sasaki A, Taylor C, Fudenberg D. Emergence of cooperation and evolutionary stability in finite populations. Nature:International weekly journal of science. 2004;428(6983):646–650. doi: 10.1038/nature02414 15071593

[59] Chen ZL, Yuan XC, Zhang XL, Cao YF. How will the Chinese national carbon emissions trading scheme work? The assessment of regional potential gains Energy Policy. 2020;137(C). doi: 10.1016/j.enpol.2019.111095

[60] Yang GS, Lu XY, Xu Y. Simulation on rate of unannounced inspection for the government investment project quality based on SD evolutionary game model. Science and Technology Management Research. 2018;38(10):212–219.

[61] Antal T, Scheuringb Ia. Fixation of strategies for an evolutionary game in finite populations. Bulletin of Mathematical Biology. 2006;68(8):1923–1944. doi: 10.1007/s11538-006-9061-4 17086490

[62] Altrock PM, Traulsen A. Determinstic evolutionary game dynamics in finite populations. Physical review E, Statistical, nonlinear, and soft matter physics. 2009;80(1pt1):011909. doi: 10.1103/PhysRevE.80.011909 19658731

[63] Taylor C, Iwasa Y, Nowak MA. A symmetry of fixation times in evoultionary dynamics. Journal of Theoretical Biology. 2006;243(2):245–251. doi: 10.1016/j.jtbi.2006.06.016 16890959 PMC2879639

[64] Liu XS, Pan QH, Kang YB, He MF. Fixation times in evolutionary games with the Moran and Fermi processes. Journal of Theoretical Biology 2015;387:214–220. doi: 10.1016/j.jtbi.2015.09.016 26416547

[65] Altrock PM, Traulsen A. Fixation times in evolutionary games under weak selection. Journal of Physics. 2009;11(1). doi: 10.1088/1367-2630/11/1/013012

[66] Zeng ZX, Sun JH, Li CY. Research on selection of low carbon strategy and game evolution of manufacturing enterprises under cloud manufacturing mode. Chinese Journal of Systems Science. 2022(03):102–107+13.

[67] Zhang M, ZHU JJ, Wang HH. Evolutionary Game Analysis of ‘‘Main Manufacturer Supplier” Collaboration Mechanism Considering Supplier’s Technology Truncation. Control and Decision. 2020:1–6. (Forthcoming).

